# Dynamic Self‐Clickable Decellularized Matrix Hydrogels for Regulating Vascularity and Enhancing Muscle Regeneration

**DOI:** 10.1002/advs.75296

**Published:** 2026-04-20

**Authors:** Van Thuy Duong, Tuba Marjan, Ngoc Ha Luong, Taimoor H. Qazi, Chien‐Chi Lin

**Affiliations:** ^1^ Weldon School of Biomedical Engineering Purdue University West Lafayette Indiana USA

**Keywords:** decellularized matrix, dynamic covalent chemistry, hydrogels, small intestine submucosa, thiol‐norbornene photoclick reaction, volumetric muscle loss

## Abstract

Decellularized matrix (dECM) derived from small intestine submucosa (SIS) has been increasingly used in tissue engineering and regenerative medicine. While dECM provides cell‐adhesive and protease‐labile sequences to support cell‐matrix interactions, its crosslinking into hydrogels has been largely limited to temperature‐induced gelation, which offers limited tunability. To address this challenge, we previously reported the synthesis of bovine decellularized SIS‐norbornene (dSIS‐NB) for crosslinking into cytocompatible thiol‐norbornene hydrogels with pro‐angiogenic and pro‐vasculogenic properties. In this study, we conducted proteomic profiling to analyze the protein compositions of bovine dSIS and dSIS‐NB. In addition to various collagens, we discovered that bovine dSIS contained significant amounts of fibrillin‐I, a glycoprotein stabilized by intra‐ and inter‐molecular disulfide bonds. We leveraged these disulfide bonds to fabricate ‘self‐clickable’ dSIS‐NB thiol‐norbornene hydrogels without the need for additional thiol‐bearing crosslinker (e.g., 4‐arm poly(ethylene glycol)‐thiol). Furthermore, we exploited thiol‐disulfide exchange in self‐clickable dSIS‐NB hydrogels to enable light‐induced spatiotemporal tuning of hydrogel stiffness and labeling of bioactive ligands. Finally, the dynamic and self‐clickable dSIS‐NB hydrogels were used as an in vitro cell culture model to study local vascular compression and as an injectable, cell‐laden matrix to treat volumetric muscle loss.

## Graphical abstract

Dynamic decellularized hydrogels are prepared using bovine decellularized small intestine submucosa (SIS) norbornene (dSIS‐NB). Bovine dSIS contained significant amounts of disulfide‐rich fibrillin‐I, enabling ‘self‐clickable’ thiol‐norbornene gelation and spatiotemporal tuning of hydrogel physicochemical properties. The self‐clickable dSIS‐NB hydrogels are used as an in vitro model for local vascular compression and as an injectable matrix for treating volumetric muscle loss.

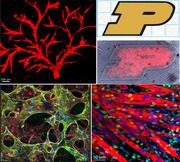

## 1 Introduction

Porcine decellularized small intestine submucosa (dSIS) has been used in various clinical applications, including wound healing, hernia repair, and bladder reconstruction [1]. Porcine dSIS is commonly formulated into powders, membranes, and sponges to fit clinical needs [2]. In addition to its long and successful history in clinical applications [2], dSIS has also been exploited as an injectable hydrogel for stem cell delivery [3]. Like most decellularized extracellular matrices (dECM), dSIS is rich in collagens, which can be physically crosslinked into hydrogels at body temperature [4]. However, temperature‐induced physical crosslinking produces soft dECM hydrogels with shear moduli (G’) of tens to hundreds of pascals [5], a narrow range that does not recapitulate the mechanical properties of most human tissues. This disadvantage can be partially mitigated by integrating dECM with other macromers to form composite hydrogels with tunable crosslinking, provided that the entrapped dECM remains in the gels [6, 7, 8]. Alternatively, native tyrosine residues can be used to photocrosslink dECM hydrogels; however, this approach is limited by the inherent availability of tyrosine [9]. Various types of dECM have been chemically modified to enable their covalent crosslinking into hydrogels, including thiolation of lung dECM [10], and methacrylation of dSIS (i.e., dSIS‐MA) [11]. However, thiolated proteins are prone to both reversible and irreversible oxidation [12]. On the other hand, chain polymerization of methacrylated macromers prolongs radical propagation and produces high‐molecular‐weight, non‐degradable hydrophobic crosslinks, causing nanoscale network heterogeneity and adverse cell‐material interactions [13]. The mechanical properties of chain‐polymerized gels are often coupled to their compositions, limiting the ability to independently tune matrix mechanics and bioactive content for mechanistic studies.

To address the challenges of chain‐polymerized hydrogels, we previously reported dSIS‐norbornene (dSIS‐NB) hydrogels crosslinked via rapid and highly tunable step‐growth photopolymerization, with 4‐arm poly(ethylene glycol)‐thiol (PEG4SH) as the crosslinker [14]. We demonstrated that orthogonally crosslinked dSIS‐NB thiol‐norbornene hydrogels were highly cytocompatible for in situ encapsulation of pancreatic cancer cells, cancer‐associated fibroblasts (CAF), and endothelial cells. When compared with thermally crosslinked soft dSIS matrices, stiffer dSIS‐NB thiol‐norbornene hydrogels supported faster and more extensive angiogenic sprouting and in situ vasculogenesis. The photoresponsive dSIS‐NB was also adapted for fabricating microgels and macroporous annealed particle (MAP) hydrogels, as well as for extrusion and digital light processing (DLP) 3D printing. More recently, we adapted dSIS‐NB hydrogels to create a functional intestinal crypt/villi epithelium, aided by DLP‐printed PEG‐based sacrificial hydrogels [15]. While these studies demonstrated many beneficial properties of orthogonally crosslinked dSIS‐NB hydrogels, the compositions of bovine dSIS and the loci of NB modification remain undetermined. In addition, prior work has explored only in vitro applications, and the therapeutic potential of this new dECM hydrogel platform has not yet been demonstrated.

In addition to expanding the potential therapeutic value of orthogonally crosslinked dSIS‐NB hydrogels, we sought to advance dSIS‐NB as a versatile biomaterial with dynamically tunable crosslinking, similar to gels crosslinked by dynamic covalent chemistry (DCC) [16]. DCC describes a group of reactions forming reversible covalent bonds, including boronic acid‐ester [17], allyl sulfide [18], disulfide [19], and hydrazone [20]. Thiol‐disulfide exchange is a particularly attractive DCC in biological applications owing to the presence of disulfide bonds in many native proteins. In principle, macromers containing multiple free sulfhydryl groups can be oxidized and polymerized into disulfide‐crosslinked hydrogels [21]. Alternatively, hydrogels can be polymerized by crosslinkers containing internal disulfide bonds [22]. In both cases, free sulfhydryl groups induce disulfide exchange, leading to macroscopic material property changes. However, most hydrogels crosslinked by a single DCC exhibit low mechanical strength due to rapidly exchanging bonds, whereas DCC with strong bonding typically forms stable gels without noticeable bond exchange under physiological conditions. The trade‐off between hydrogel stiffness and reversibility of DCC can be mitigated by incorporating a dual‐bonding strategy in which the hydrogels are crosslinked by both irreversible and reversible covalent bonds. For example, the Anseth group developed adaptable fast‐relaxing hydrogels by incorporating both permanent bonds formed by strain‐promoted azide‐alkyne cycloaddition (SPAAC) and reversible boronate‐ester bonds [23]. Our groups have also adapted this dual bonding strategy to creating gelatin‐norbornene‐boronate (GelNB‐BA) hydrogels with modularly tunable stiffness and viscoelasticity [24]. Nonetheless, it is unclear whether the orthogonally crosslinked dSIS‐NB hydrogels can be adapted to exhibit dynamically tunable properties.

In this study, we conducted proteomic profiling to characterize the compositions of bovine dSIS and dSIS‐NB, as well as the locations of NB modification. We evaluated the thiol content in bovine dSIS samples and demonstrated that dSIS‐NB can be photocrosslinked into hydrogels in the presence of lithium phenyl‐2,4,6‐trimethylbenzoylphosphinate (LAP) as a photoinitiator, but without additional thiol‐crosslinker (e.g., PEG4SH). The LAP‐mediated self‐click photocrosslinking was attributed to the inherent disulfide bonds in bovine dSIS. As no additional crosslinker is needed for the gelation, we refer to this unique crosslinking scheme as ‘self‐clickable’ thiol‐norbornene crosslinking. Furthermore, we leveraged the rich disulfide bonds in bovine dSIS‐NB to dynamically tune hydrogel stiffness and angiogenic properties to control matrix vascularity. Finally, the therapeutic potential of self‐clickable and injectable dSIS‐NB hydrogels for supporting tissue repair was evaluated using a mouse model of volumetric muscle loss (VML).

## 2 Results and Discussion

### 2.1 Self‐Click Crosslinking of dSIS‐NB Hydrogels

**FIGURE 1:**
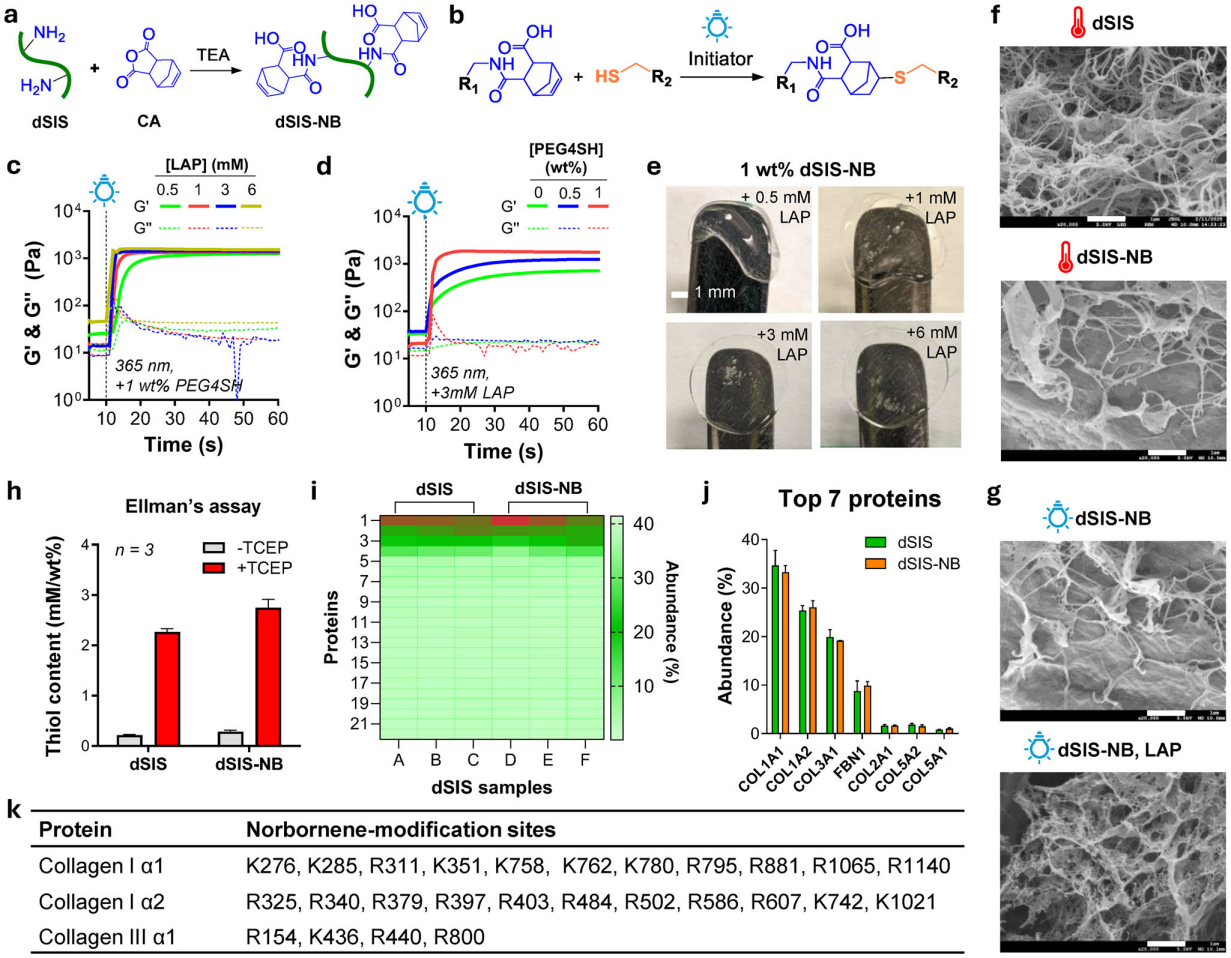
Self‐clickable dSIS‐NB hydrogel. (a) Scheme of dSIS‐NB synthesis. (b) Scheme of thiol‐norbornene photo‐click gelation. (c) In situ photo‐rheometry to monitor the crosslinking of 1 wt.% dSIS‐NB and 1 wt.% PEG4SH at different LAP concentrations (n = 3, error bars not shown for graph clarity). (d) In situ photo‐rheometry to monitor the crosslinking of 1 wt.% dSIS‐NB and various PEG4SH with 3 mm LAP (n = 3, error bars not shown for graph clarity). (e) Photographs of self‐clickable dSIS‐NB hydrogels crosslinked with different LAP concentrations. (f) SEM images of thermally crosslinked dSIS/dSIS‐NB gels. (g) SEM images of dSIS/dSIS‐NB crosslinked by light irradiation (scales: 1 µm). All thiol‐norbornene gelation was initiated by 365 nm light at 8 mW/cm^2^. (h) Quantification of free thiol contents in bovine dSIS and dSIS‐NB samples before and after reduction with TCEP (n = 3, Mean ± SEM). (i) Heat map protein abundance from proteomic profiling of bovine dSIS and dSIS‐NB. Three independent batches of dSIS and dSIS‐NB were analyzed and 23 proteins were consistently identified from all six samples. (j) Abundance of the top 7 proteins from the proteomic profiling (n = 3, Mean ± SEM). (k) Amino acid residues of norbornene modification on dSIS‐NB.

dSIS‐NB was synthesized via reacting primary amines on bovine dSIS with carbic anhydride (Figure 1a). We determined the NB substitution to be approximately 10% across three independent reaction batches (∼0.28 mm norbornene in 1 wt.% dSIS‐NB; Table S1). dSIS‐NB could be crosslinked with thiol‐bearing crosslinker (e.g., PEG4SH) into covalent hydrogels via LAP‐mediated thiol‐norbornene photo‐click reaction (Figure 1b) [14, 25]. As photoinitiator LAP has a high molar extinction coefficient at 365 nm (ε = 218 m ^−1^cm^−1^) [26], crosslinking of 1 wt.% dSIS‐NB and 1 wt.% PEG4SH at 365 nm reached gel point after 2 to 5 s of light exposure and achieved completion within 30 s even at a low light intensity (8 mW/cm^2^) and a very low LAP concentration (0.5 mm) (Figure 1c). At 405 nm light, where LAP only has a lower molar absorbability (ε = 25 m ^−1^cm^−1^), dSIS‐NB gelation could still be achieved in under 30 s using a moderate LAP concentration (1 to 6 mm) (Figure S1). Orthogonal thiol‐norbornene gelation is typically achieved by using an NB‐modified polymer and a thiol‐bearing crosslinker under varying stoichiometric ratios of thiol to norbornene, with the maximum degree of crosslinking achieved at a thiol‐to‐norbornene ratio of 1. In 1 wt.% dSIS‐NB, the norbornene content was about 0.28 mm (Table S1). On the other hand, the free thiol content on 1 wt.% of PEG4SH (10 kDa) was 4 mm, which was 14‐fold higher than NB concentration on dSIS‐NB. Even with excessive thiols, dSIS‐NB was rapidly crosslinked into hydrogels with a stable network within 5 to 50 s (Figure 1c; Figure S1). Reducing PEG4SH to 0.5 wt.% did not alter gel point but lowered the plateau shear storage modulus (G’) from ∼1700 Pa to ∼1200 Pa (Figure 1d). Interestingly, crosslinking of dSIS‐NB in the absence of PEG4SH still yielded a gel point of ∼2.5 s and reached plateau moduli of ∼700 Pa after 30 s of light exposure (Figure 1d). The PEG4SH‐independent gelation of dSIS‐NB was visualized via photocrosslinking 1 wt.% dSIS‐NB with different LAP concentrations. At 0.5 mm LAP, a soft hydrogel was formed after 2 min of 365 nm light exposure. At 3 and 6 mm of LAP, the hydrogel discs were stiffer and not deformed when placed on a spatula (Figure 1e). We further evaluated the nanostructure of bovine dSIS and dSIS‐NB hydrogels via scanning electron microscopy (SEM). As expected, thermally gelled dSIS and dSIS‐NB exhibited typical nanoporous and fibrous structures (Figure 1f). 365 nm light irradiation alone did not cause a noticeable difference in the nanofibrous structure of dSIS‐NB matrix. Interestingly, LAP and light crosslinked dSIS‐NB hydrogel maintained a nanofibrous structure, but the fibers were noticeably thinner and formed a mesh‐like network (Figure 1g). When dSIS‐NB was crosslinked into hydrogels with PEG4SH, the matrix appeared like a typical chemically crosslinked hydrogel, although some fibrous structure was ‘buried’ within the smoother matrix (Figure S2).

### 2.2 Proteomic Profiling of dSIS and dSIS‐NB

To understand the mechanism of self‐click crosslinking of dSIS‐NB, we performed Ellman's assay with non‐reduced dSIS and dSIS‐NB and found a small but measurable free sulfhydryl group in dSIS and dSIS‐NB (∼0.21 to 0.28 mm, respectively. Figure 1h). This amount was almost equivalent to the NB content on dSIS‐NB, suggesting that the self‐click crosslinking of dSIS‐NB (i.e., in the absence of PEG4SH) was facilitated, at least in part, by the free sulfhydryl group on bovine dSIS. Interestingly, treating dSIS and dSIS‐NB samples with a thiol‐free reducing agent tris(2‐carboxyethyl)phosphine (TCEP) [27, 28], led to substantial increases in free sulfhydryl contents on both samples (Figure 1h). Specifically, TCEP treatment led to ∼10‐fold increase in free thiol contents in dSIS‐NB (e.g., ∼ 2.7 mm of free thiols per wt.% dSIS‐NB). To identify the source of these additional thiol groups, we conducted mass‐spec proteomics to profile six bovine dSIS samples, including three independent batches of dSIS and dSIS‐NB. We identified 102 proteins at least once among the 6 samples. Only 22 proteins appeared consistently in all 6 (Figure 1i), and the top 4 proteins (collagen I α1, collagen I α2, collagen III α1, and fibrillin 1) made up more than 90% of all proteins (Figure 1j).

From the proteomic results, we found that norbornene conjugation did not change dSIS protein compositions (Figure 1i,j). Moreover, NB modifications were found on lysine and arginine residues of Type I and Type III collagens (Figure 1k), and none on fibrillin or any of the other less abundant proteins. We further studied the amino acid composition and found that collagen only contains a negligible amount of cysteine residues (0.3%–2%) [29, 30]. On the other hand, fibrillin 1, which accounts for ∼10% of total proteins in dSIS samples, harbors ∼12% of cystine residues in its amino acid composition (Table S2). Therefore, we concluded that fibrillin was mainly responsible for the self‐click gelation of dSIS‐NB in the absence of exogenously added thiol‐crosslinker (Figure 1d,e). The presence of disulfide‐rich fibrillin appeared to be unique to bovine dSIS, as little to no fibrillin was found in porcine dSIS samples [11]. In summary, we discovered bovine dSIS‐NB as a novel macromer for forming orthogonally crosslinkable dECM hydrogels without the need for an additional thiol‐bearing crosslinker, which is typically required in thiol‐norbornene hydrogel crosslinking. We refer to this new hydrogel as ‘self‐clickable’ dECM hydrogels.

### 2.3 Rheological Properties of Self‐Clickable dSIS‐NB Hydrogels

**FIGURE 2:**
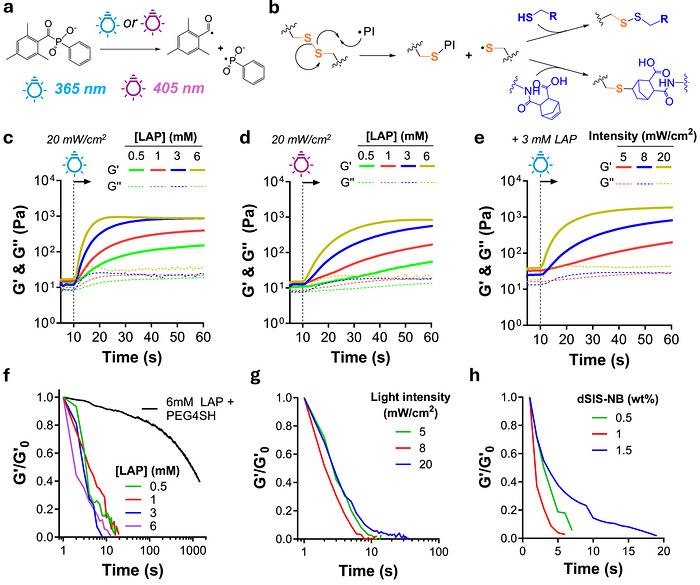
Gelation and viscoelasticity of self‐clickable dSIS‐NB hydrogel. (a) Schematic of photolysis of LAP induced by 365 or 405 nm light irradiation. (b) Radical species generated from LAP (▪PI) break disulfide bonds within dSIS‐NB to form thiyl radicals, which can either react with norbornene groups on the dSIS‐NB backbone to form thioether bonds, or undergo disulfide exchange. (c‐e) in situ photo‐rheometry of gelation using 1 wt.% dSIS‐NB with varying LAP concentrations under 365 nm light (c), 405 nm light (d), or with 3 mm LAP under 365 nm light and varying light intensities (e) (n = 3, error bars not shown for graph clarity). (f‐h) Stress‐relaxation of self‐clicked dSIS‐NB hydrogels formed by varying conditions, including changing LAP concentrations (f), varying light intensities (g), or varying dSIS‐NB concentrations (h). Gelation in was performed using 365 nm light at 8 mW/cm^2^. Gels in g and h were crosslinked by 3 mm LAP (n = 3, error bars not shown for graph clarity).

We reasoned that the high abundance of disulfide bonds in fibrillin could be leveraged to adjust hydrogel stiffness through a light/radical‐initiated multiple fragmentation and disulfide exchange mechanism, akin to that reported in purely disulfide‐crosslinked PEG‐based hydrogels [21]. The crosslinking of self‐clickable dSIS‐NB hydrogel is initiated by light irradiation (365 or 405 nm), which generates two radical species from one type I photoinitiator (i.e., LAP. Figure 2a). These radicals attack the disulfide bond within fibrillins in dSIS‐NB, generating thiyl radicals (Figure 2b) that either react with norbornenes on the dSIS‐NB backbone to form thioether bonds (Figure 2b) or undergo disulfide exchange across different fibrillin molecules. This mechanism was validated by crosslinking dSIS‐NB in the presence of varying LAP concentrations using light at 365 nm (Figure 2c) or 405 nm (Figure 2d), as well as with 365 nm light at different intensities (Figure 2e). Unlike the rapid gelation of dSIS‐NB with PEG4SH as an additional crosslinker (Figure 1c), the crosslinking of self‐clickable dSIS‐NB hydrogel did not reach completion within 50 s of light irradiation at lower LAP concentrations (0.5 and 1 mm at 365 nm; 0.5, 1, and 3 mm at 405 nm) or lower light intensity (5 and 8 mW/cm^2^ for 365 and 405 nm light, respectively). The slower gelation at 405 nm was not surprising, as LAP has a lower molar extinction coefficient at this wavelength, leading to slower generation of radical species from LAP [26]. Consequently, the relatively slower initiation rate led to gradual production of thiyl radicals and slower increases in G’. The slower gelation kinetics of self‐clickable dSIS‐NB hydrogels may be more beneficial, as low radical concentrations during gelation could minimize potential damage to the encapsulated cells [31]. It is worth noting that the ‘slower’ gelation of self‐clicked dSIS‐NB hydrogels, as described above, was relative to the ultrafast crosslinking when PEG4SH was added (Figure 1c). The speed of self‐click dSIS‐NB gelation is still fast compared to thermally gelled dSIS, as gelation can reach plateau modulus in as fast as ∼10 s (Figure 2c).

Using strain‐sweep tests, we further assessed the crosslinking of self‐clickable dSIS‐NB hydrogels. At 1 wt.% dSIS‐NB, G’ was tuned from ∼200 to ∼1,500 Pa by adjusting LAP concentration (0.5 to 6 mm, Figure S3a). At 3 mm LAP, self‐clickable dSIS‐NB hydrogel stiffness increased nearly two‐fold by increasing light intensity from 5 to 8 mW/cm^2^ (Figure S3b). Further increasing light intensity to 20 mW/cm^2^ did not yield stiffer gels. Additionally, increasing dSIS‐NB concentration from 0.5 to 1.5 wt.% resulted in a significant increase in G', from approximately 450 to 1800 Pa (Figure S3c). 365 nm light at 8 mW/cm^2^ was used as the optimal light intensity for subsequent hydrogel crosslinking.

Matrices with nanofibrous structures tend to exhibit high viscoelasticity or fast relaxation, as the fibrous network dissipates energy under deformation [32]. We measured the stress‐relaxation profiles (i.e., G’/G’_0_ changes as a function of time) of self‐clickable dSIS‐NB hydrogels crosslinked with 0.5, 1, 3, and 6 mm LAP, and compared that with dSIS‐NB crosslinked by PEG4SH gels using 6 mm LAP (Figure 2f). While the shear moduli of self‐clickable dSIS‐NB hydrogels scaled with LAP concentration from 0.5 to 6 mm (Figure 2c; Figure S3a), all self‐clickable dSIS‐NB hydrogels exhibited fast stress‐relaxation profiles, with relaxation halftimes around 3 to 10 s (Figure 2f). In contrast, the stress‐relaxation of PEG4SH‐crosslinked dSIS‐NB hydrogels was noticeably slower, with relaxation halftime at around 1000 s. The fast relaxation of self‐clickable dSIS‐NB hydrogels was also observed in gels crosslinked with different light intensity (Figure 2) and with different dSIS‐NB content (Figure 2h). In summary, the self‐clickable dSIS‐NB hydrogels exhibited tunable crosslinking while maintaining the characteristic fast‐relaxation of nanofibrous dECM matrices, which is typically challenging to achieve with chemically modified dECM hydrogels [11, 33].

### 2.4 Dynamic Stiffening, Softening, and Patterning of Self‐Clickable dSIS‐NB Hydrogels

**FIGURE 3:**
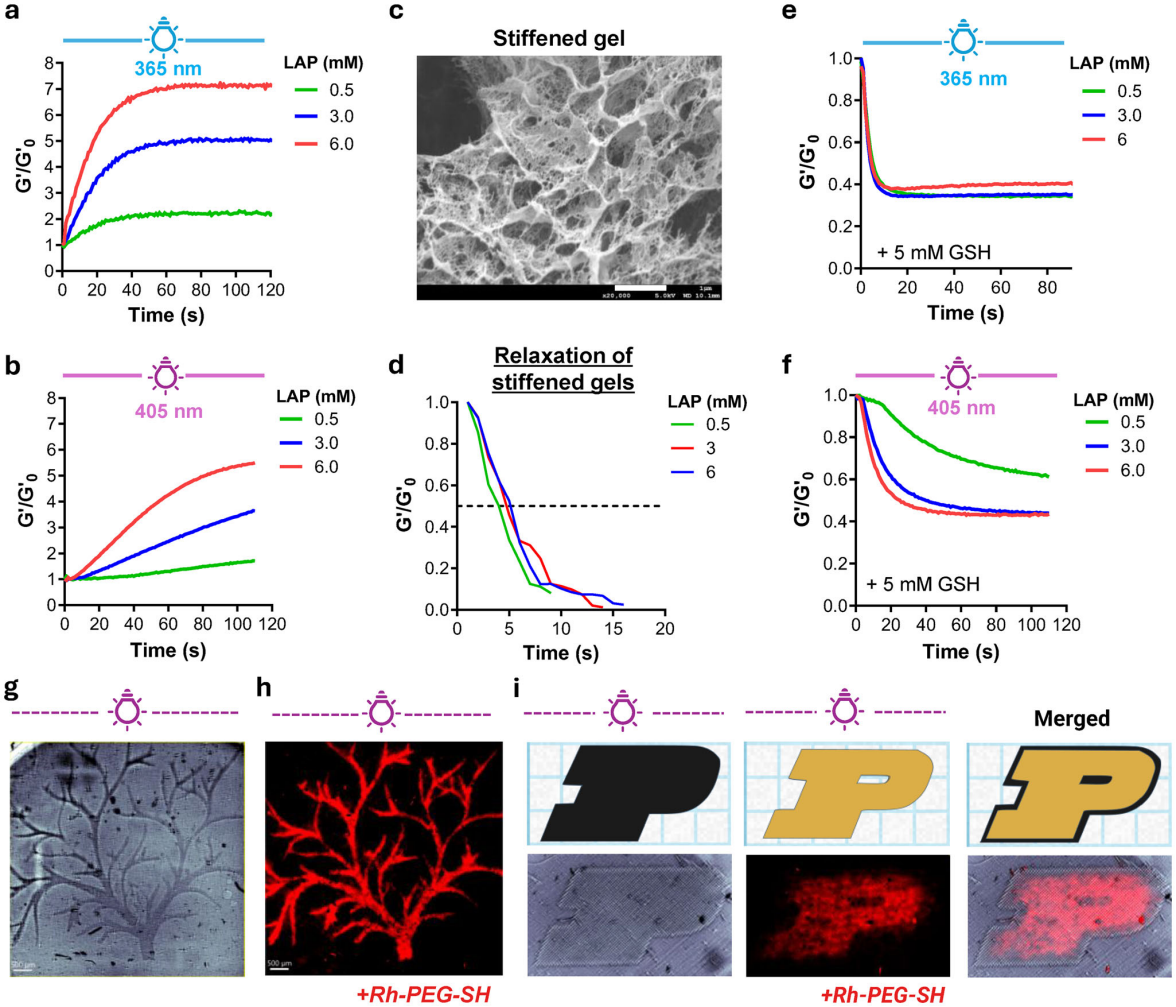
Radical‐mediated dynamic stiffening, softening, & patterning of self‐clicked dSIS‐NB hydrogels. (a, b) in situ photo‐rheometry of light‐induced stiffening of self‐clicked dSIS‐NB hydrogels by supplying additional LAP and secondary light exposure at 365 nm (a) or 405 nm (b) (n = 3, error bars not shown for graph clarity). (c) SEM image of a self‐clicked dSIS‐NB hydrogel stiffened by 6 mm LAP and 365 nm light (8 mW/cm^2^, 2 min). (d) Stress‐relaxation profiles of self‐clicked dSIS‐NB hydrogels stiffened by varying LAP concentrations and 365 nm light (n = 3, error bars not shown for graph clarity). (e, f) in situ photo‐rheometry of light‐induced softening of self‐clicked dSIS‐NB hydrogels by supplying additional LAP and secondary light exposure at 365 nm (e) or 405 nm (f) (n = 3, error bars not shown for graph clarity). Soft gels in (a‐d) and stiff gels in (e‐f) were crosslinked by 1 wt.% dSIS‐NB with 0.5 and 6 mm LAP, respectively (365 nm, 8 mW/cm^2^, 2 min). 5 mM GSH was added for gel‐softening. (g, h) Spatially controlled stiffening (g) and ligand immobilization (h) in self‐clicked dSIS‐NB hydrogels. Gels were formed by 0.5 and 6 mm LAP, respectively. (i) Sequential stiffening and patterning on the same gel with Purdue ‘*P*’ logo. Gel was formed by 0.5 mm LAP, 0.5 mm tartrazine, and 405 nm light irradiation at 34 mW/cm^2^ for 2 min.

After demonstrating the self‐click crosslinking of dSIS‐NB, we sought to further exploit fibrillin's disulfide bonds to stiffen and soften the dSIS‐NB gels dynamically. A soft hydrogel was pre‐formed using 1 wt.% dSIS‐NB with 0.5 mm LAP and exposed to 365 nm light (8 mW/cm^2^) for 2 min, resulting in an initial storage modulus of approximately 200 Pa. The soft hydrogels were incubated in PBS containing additional LAP, followed by light irradiation at 365 or 405 nm to generate more thiyl radicals that participated in disulfide exchange reactions, thereby increasing gel stiffness. Specifically, 365 nm light irradiation in the presence of 0.5‐, 3‐, or 6‐mm LAP increased gel stiffness by approximately 2‐, 4‐, or 7‐fold within 20 s, respectively (Figure 3a). The stiffening time was prolonged to approximately 100 s when the same formulation was exposed to 405 nm light (Figure 3b). Additionally, exposure to 365 nm light resulted in a higher final G’ compared to 405 nm light exposure. Comparing SEM images in Figures 3c and 1g, there was no observable difference between the soft (Figure 1g, using 3 mm LAP) and stiffened self‐clickable dSIS‐NB gels (Figure 3c) in their nanofibrous structures. The viscoelasticity of stiffened self‐clickable dSIS‐NB gels also did not change (Figure 3d) compared with the data shown in Figure 2f, and the stress‐relaxation halftimes remained around 3 to 10 s. This was consistent with the high viscoelasticity of stiff (Figure 2f) and stiffened (Figure 3d) self‐clickable dSIS‐NB gels.

To induce dynamic softening, dSIS‐NB hydrogels were exposed to 365 or 405 nm light in the presence of LAP and monothiol molecules such as glutathione (GSH), rhodamine‐PEG‐thiol (Rh‐PEGSH), or thiolated peptides. These mono‐thiol reagents facilitate termination of the disulfide exchange reactions, yielding softer gels with pendant ligand immobilization. This was demonstrated by softening a stiff dSIS‐NB hydrogel (G’ ∼1500 Pa) crosslinked by 1 wt.% dSIS‐NB with 6 mm LAP (365 nm light for 2 min, 8 mW/cm^2^). The gel was incubated in PBS containing 5 mm GSH and LAP before in situ photo‐rheometry (Figure 3e). The gel stiffness rapidly decreased by approximately 62% within a few seconds under 365 nm light, regardless of LAP concentration. The softening time was extended to approximately 10–60 s when the same gel formulations were exposed to 405 nm light (Figure 3f). The softening effect reached a plateau as a portion of the crosslinks were non‐degradable thioether bonds formed between norbornene and thiol groups [34].

We reasoned that the light/disulfide‐mediated stiffening/softening of the dSIS‐NB hydrogels can be achieved spatiotemporally, similar to other photo‐tunable hydrogels. To demonstrate this concept, we created a complex tree‐like branching pattern from the TinkerCAD website. The pattern was irradiated over a DLP‐printed soft dSIS‐NB hydrogel (6 mm LAP, 0.5 mm Tartrazine, 405 nm. G’ ∼ 230 Pa). The exposed branching areas were stiffened and became darker (Figure 3g). Parallel stiffening experiments show that the G’ of stiffened gel reached 1.2 kPa. To demonstrate spatially‐controlled softening and ligand tethering, we incubated another stiff self‐clicked dSIS‐NB hydrogel (G’ ∼1600 Pa), in 5 mm rhodamine‐PEGSH (Rh‐PEGSH), 6 mm LAP, and 0.5 mm Tartrazine, followed by light irradiation through the tree pattern. A red tree‐branch pattern appeared on the hydrogel after light irradiation, demonstrating the spatial conjugation of Rh‐PEGSH (Figure 3h). Parallel softening experiments show that the G’ of the softened gels was ∼950 Pa. Finally, we achieved sequential and spatial stiffening *and* softening within the same self‐clickable dSIS‐NB gel using the same process described above, creating a Purdue University *“P”* logo with a stiffened outer *P* and a softened inner *P* (Figure 3i). Of note, the small *P* overlaid the large *P* to form a Purdue University *“P”* logo, where the red area was initially soft, then stiffened, and then softened again.

### 2.5 Self‐Clickable dSIS‐NB Hydrogels Promote Vascularization and Angiogenic Sprouting

**FIGURE 4:**
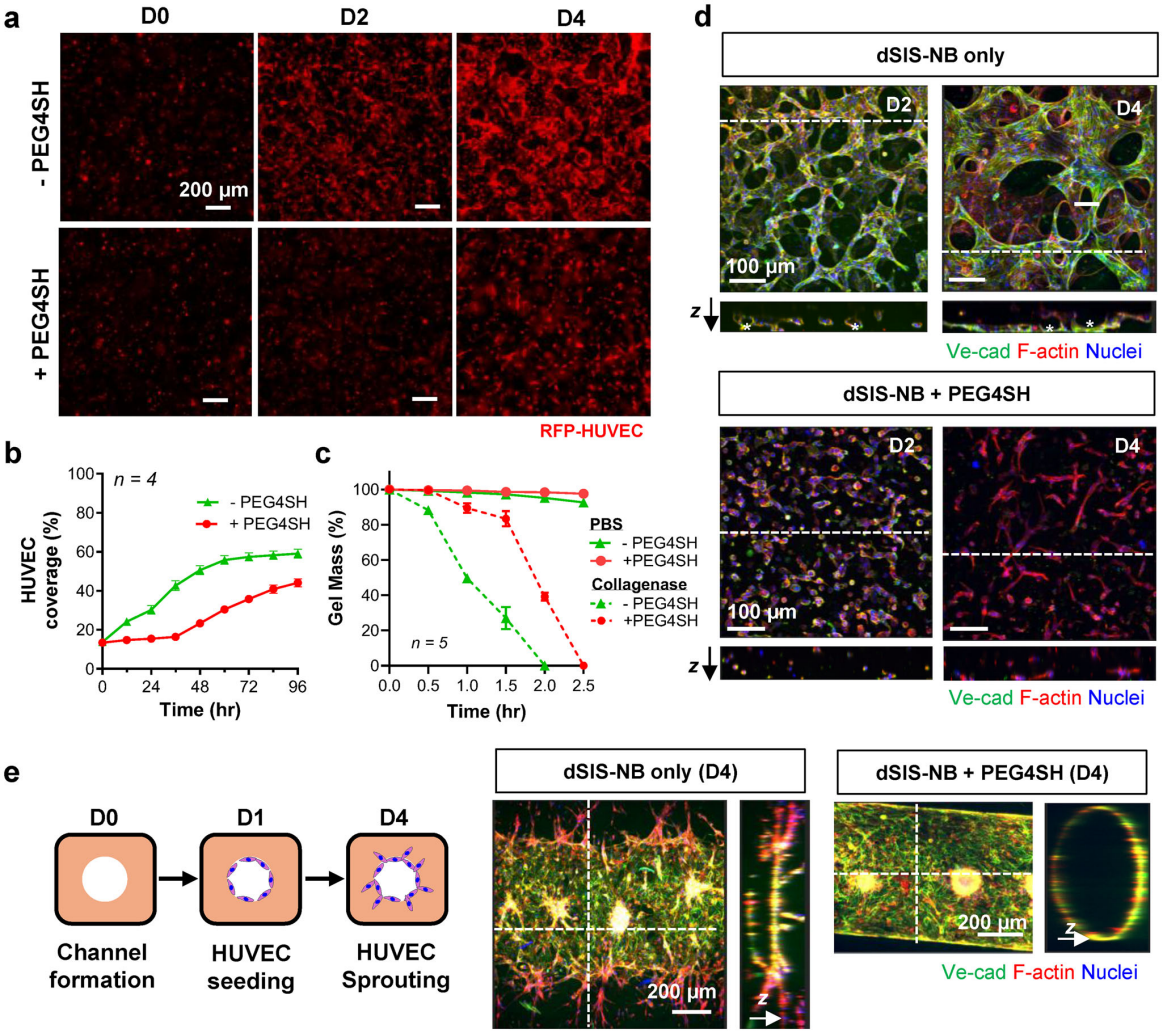
Vascularization of self‐clickable dSIS‐NB hydrogels. (a) Time‐lapse imaging of network formation of RFP‐HUVECs encapsulated within self‐clickable dSIS‐NB and PEG4SH‐crosslinked dSIS‐NB hydrogels. (b) Cell coverage over 96 h using live cell tracking images (n = 4, Mean ± SEM). (c) in vitro proteolytic degradation of 1 wt.% dSIS‐NB hydrogels crosslinked with or without 1 wt.% PEG4SH. All gels were crosslinked by 6 mm LAP under 365 nm light at 5 mW/cm^2^ (n = 5, Mean ± SEM). (d) Vascular network formation from encapsulated HUVECs in self‐clickable dSIS‐NB hydrogels or PEG4SH‐crosslinked dSIS‐NB hydrogels. (f) Angiogenic sprouting from endothelial vessels into self‐clicked dSIS‐NB hydrogels or PEG4SH‐crosslinked dSIS‐NB hydrogels.

To demonstrate the potential of using self‐clickable dSIS‐NB hydrogels in tissue engineering, we first encapsulated red fluorescent protein‐tagged human umbilical vascular endothelial cells (RFP‐HUVECs) in dSIS‐NB hydrogels crosslinked with 3 mm LAP in the absence or presence of PEG4SH. Live‐cell tracking showed that the encapsulated RFP‐HUVECs proliferated and formed networks significantly faster in self‐clickable dSIS‐NB gel than in PEG4SH‐crosslinked hydrogels (Figure 4a; Video S1). In the self‐clickable dSIS‐NB gels, RFP‐HUVEC‐covered area increased from approximately 17% to 30% within 24 h, reaching 50% after 48 h and remaining nearly stable until 96 h (Figure 4b). On the other hand, a lag phase in cell area was observed in the dSIS‐NB/PEG4SH gel during the first 36 h, followed by a gradual increase to 38% by 96 h. The delay in endothelial network formation in PEG4SH‐crosslinked gels may be due to reduced matrix degradation by cell‐secreted proteases, such as matrix metalloproteinases (MMPs). Hence, we assessed the proteolytic degradation of dSIS‐NB hydrogels crosslinked either in the absence (i.e., self‐click crosslinking) or presence of PEG4SH. As shown in Figure 4c, exogenous collagenase I treatment led to faster reduction of gel mass in self‐clicked dSIS‐NB hydrogels than those crosslinked by PEG4SH, suggesting that self‐clickable dSIS‐NB hydrogels were more prone to proteolytic degradation.

Next, non‐fluorescent HUVECs were encapsulated for assessing vascular network formation. In 2 days, HUVECs in the self‐clickable dSIS‐NB gels formed extensive interconnected vascular networks with small lumen structures (∼20 µm in diameter), whereas HUVECs in the dSIS‐NB/PEG4SH gels had only begun to spread and did not form continuous networks or lumen structures (Figure 4d). In the self‐clickable dSIS‐NB gels, branches of the vascular network merged to form larger channels with some diameters larger than 100 µm after 4 days. In contrast, HUVECs in the dSIS‐NB/PEG4SH gels fused and elongated without forming lumen structures. Vessel length density (VLD) was quantified using z‐stack confocal images from a stack of images with a total thickness of 200 µm and an imaging area of 0.41 mm^2^ (Figure S4a). On day 2, VLD in the self‐clickable dSIS‐NB gel was 2.2 times higher than that in the dSIS‐NB‐PEG4SH gel, and the difference increased to about 3.2 times on day 4. Image analysis also showed that VE‐cadherin expression in HUVECs within the self‐clickable dSIS‐NB gels was significantly higher than in the dSIS‐NB‐PEG4SH gels on both day 2 and day 4 (Figure S4b). To assess the angiogenic properties of self‐clicked dSIS‐NB hydrogels, we fabricated hydrogels with hollow channels for seeding HUVECs (Figure 4e). In the self‐clickable dSIS‐NB gel, HUVECs formed a monolayer on the channel surface and began to protrude into the bulk gel after 24 h (Figure 4e; Figure S5). On day 4, the sprouts within the self‐clickable dSIS‐NB gel were almost twice as long (400 µm) as on day 2. On the other hand, no protrusions were observed in the PEG4SH‐crosslinked dSIS‐NB gels in 4 days (Figure 4e; Figures S5 and S6a). The number of sprouting vessels from the main channel was also significantly higher in the self‐clickable dSIS‐NB gel compared to the dSIS‐NB‐PEG4SH gel on both day 2 and day 4 (Figure S6b).

Vascularization of tissue‐engineered scaffolds is an ongoing challenge [35, 36, 37]. In our previous study, we demonstrated that encapsulated HUVECs formed vascular networks within 3 to 5 days in 0.8 wt.% dSIS‐NB gels crosslinked with 0.025 wt.% and 0.8 wt.% PEG4SH, respectively [14]. The self‐clickable dSIS‐NB gel in the current study significantly accelerated the network formation, achieving a primitive lumenized vascular network within 2 days (Figure 4; Video S1). Self‐clicked dSIS‐NB hydrogels also support rapid angiogenic sprouting (< 24 h) from confluent HUVECs formed in the hydrogel channel (Figure 4e; Figure S5). Others have shown that porcine SIS gels induced better neovascularization than type I collagen gels, faster vessel sprouting, and increased angiogenesis in vivo [38]. They also demonstrated that the SIS gels effectively activated key angiogenic genes (KDR, Notch1, Ang2) in seeded HUVECs. In our study, because both hydrogel groups contained the same dSIS‐NB content (1 wt.%), differences in vascular network formation and angiogenic sprouting could be attributed to faster proteolytic degradation (Figure 4c) of the self‐clickable dSIS‐NB hydrogels relative to the PEG4SH‐crosslinked counterparts. Furthermore, PEG4SH‐crosslinked dSIS‐NB hydrogels had higher initial stiffness (G’ ∼ 2.1 kPa) and slower stress‐relaxation than those of self‐clicked dSIS‐NB hydrogels (Figure 2f), resulting in slower vascular network formation of HUVEC [14]. However, the stiffness of the unmodified dSIS matrix was below 100 Pa, yet significantly slower vascularization speed was observed in our prior study [14], suggesting that self‐clickable dSIS‐NB hydrogels may provide a better mechanical environment to maximize the vascularization speed. In addition to matrix mechanics, protein composition in bovine dSIS may contribute to the pro‐angiogenic effect of dSIS‐NB hydrogels. The top 4 proteins in dSIS and dSIS‐NB are collagen I α1, collagen I α2, collagen III α1, and fibrillin I (Figure 1i,j). We reasoned that the prominent presence of fibrillin I (∼10%) in bovine dSIS may contribute to enhanced vascularization of HUVECs by activating pro‐angiogenic integrin α5β1 [39, 40, 41], while collagen I and III do not activate α5β1. Future studies are needed to elucidate the molecular signaling pathways underlying the enhanced angiogenesis and vascularization properties of bovine dSIS‐NB hydrogels.

### 2.6 Self‐Clickable and Dynamic dSIS‐NB Hydrogels to Regulate Matrix Vascularity

**FIGURE 5:**
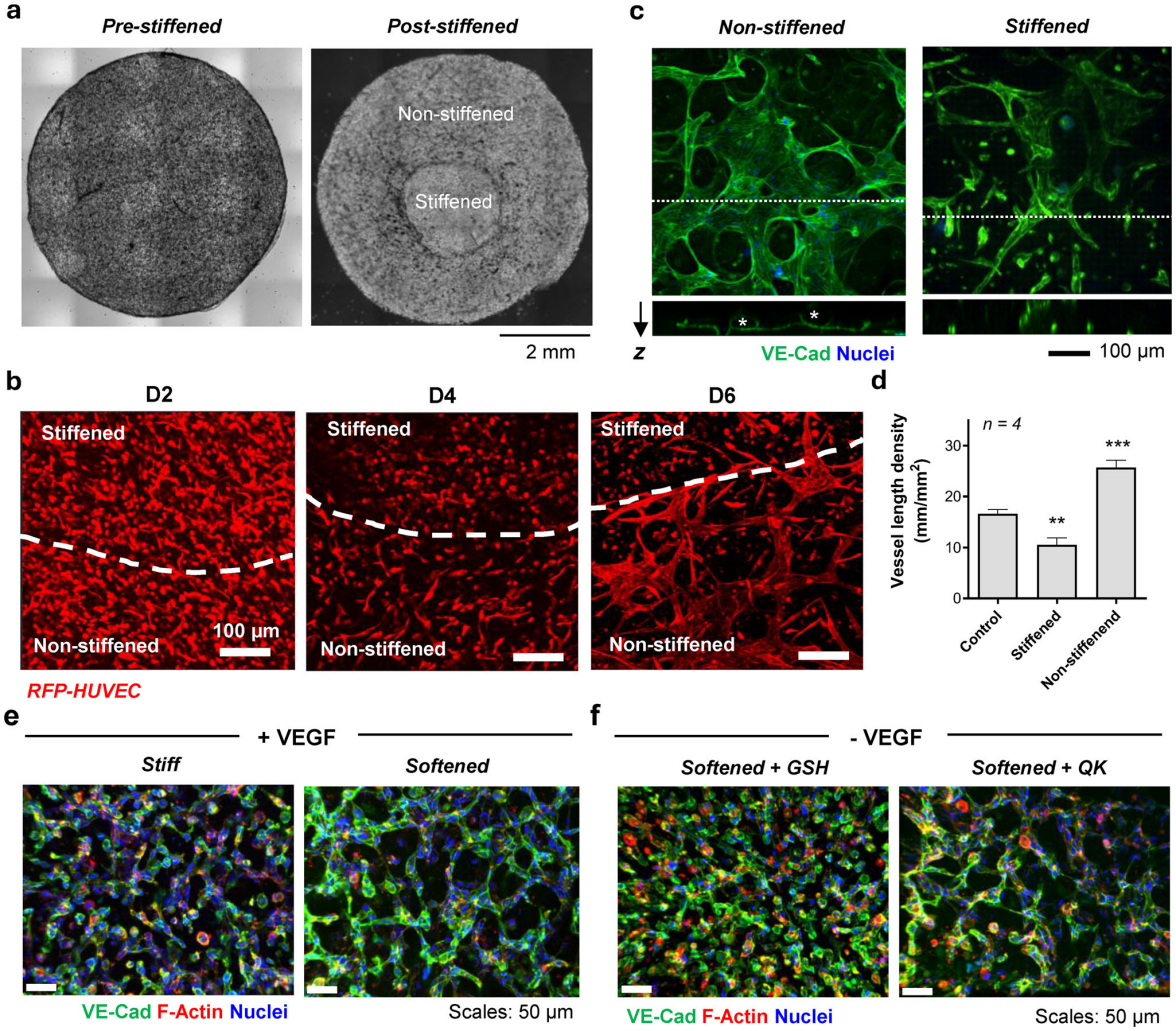
Dynamic self‐clicked dSIS‐NB hydrogels to regular matrix vascularity. (a) Brightfield images of a self‐clicked HUVEC‐laden dSIS‐NB hydrogel with the central area stiffened by photopatterning. (b) Vascular network formation from encapsulated RFP‐HUVECs at the boundary of the circular stiffened area. (c) Immunofluorescence staining of VE‐Cadherin on D6. (d) Quantification of vessel length density of RFP‐HUVEC networks within stiffened and non‐stiffened areas on D6 compared with D2 (control) (n = 3, Mean ± SEM. **p < 0.01, ***p < 0.001 compared to control group. One‐way ANOVA). (e) Controlling vascularity of HUVECs encapsulated in stiff or photo‐softened dSIS‐NB hydrogels in VEGF‐containing media. (f) Controlling vascularity of HUVECs encapsulated in softened dSIS‐NB hydrogels in the absence of VEGF. Vascularity was rescued via immobilizing angiogenic QK peptide (50 µm) in self‐clicked dSIS‐NB hydrogels.

After demonstrating the dynamic tunability (Figure 3) and vasculogenic/angiogenic potential of self‐clickable dSIS‐NB hydrogels (Figure 4), we sought to regulate matrix vascularity by dynamic tuning of endothelial cell‐laden self‐clickable dSIS‐NB hydrogels. This is particularly important in tumor matrix biology, where high desmoplastic reaction could stiffen the tumor matrix and reduce vascularity [42, 43]. We first encapsulated HUVECs in 2 wt.% self‐clicked dSIS‐NB hydrogels with an initial storage modulus of ∼0.9 kPa. On day 2 post‐encapsulation, the HUVEC‐laden self‐clicked dSIS‐NB hydrogel was incubated in media containing 3 mm LAP for 10 min, followed by 2 min of 365‐nm light exposure at the center of the hydrogel through a circular photomask. The stiffened area was clearly evident in the gel photographs before and after stiffening (Figure 5a). The storage moduli of the stiffened area increased to approximately 2.2 kPa. As expected, HUVECs formed interconnected networks within the soft self‐clickable dSIS‐NB gels (Figure 5b, D2), and there was no visible difference between stiffened and non‐stiffened areas. However, at D4 (2 days after stiffening), HUVECs formed longer micro vessels in non‐stiffened areas and appeared more rounded in stiffened areas (Figure 5b, D4). Two more days later (i.e., D6), the cellular network within the non‐stiffened area was mature and formed lumen structures (Ø > 100 µm) more than that in the stiffened area (Figure 5b, D6 and Figure 5c). VLD on D6 was significantly lower in the stiffened areas (Figure 5d).

Local matrix stiffening leads to vessel compression, whereas softening of the stiff matrix could restore vasculature. To demonstrate this concept, we encapsulated HUVECs in stiff self‐clicked dSIS‐NB hydrogels (G’ ∼ 1.4 kPa). After 1 day of culture, the cell‐laden hydrogels were incubated in GSH (1 mm) and LAP (3 mm), then exposed to 365 nm light (8 mW/cm^2^) for 2 min to induce softening. HUVECs in the GSH‐softened gel formed more interconnected networks after 2 days of culture in VEGF‐containing media (Figure 5e), with VLD reaching approximately 24.3 mm/mm^2^ (Figure S7). This result indicates that dynamic softening promoted vascular network formation of HUVECs. We also softened other HUVEC‐encapsulated gels with the same formulation and cultured them in VEGF‐free media. VEGF‐derivation led to reduced HUVECs spreading (Figure 5f), with VLD of just 13.7 mm/mm^2^ (Figure S7). When dSIS‐NB hydrogels were softened in the presence of VEGF‐mimetic QK peptide (with an additional cysteine residue to permit thiol‐disulfide exchange), the vascular network was restored rapidly, with VLD reaching ∼27.1 mm/mm^2^ after two days (Figure 5f; Figure S7). These results indicate that the immobilized QK peptide can overcome VEGF deprivation. Indeed, increasing the concentration of immobilized QK peptide led to robust network formation (Figure S8a). Note that the gel stiffness decreased moderately from ∼1300 to 800 Pa as the QK peptide concentration increased from 0.05 to 1 mm (Figure S8b). The VLD of the QK‐immobilized gels was approximately 1.63 times greater than in gels without QK peptide (Figure S8c). When QK peptide concentration was further increased from 0.05 to 1 mm, the HUVEC coverage increased from approximately 32% to 40% (Figure S8d).

Several research groups have used dECM for vascularization. For example, Choi et al. encapsulated human skeletal muscle cells and HUVECs within 3D‐printed dECM hydrogels to develop pre‐vascularized muscle constructs for the treatment of volumetric muscle loss (VML) [44]. These constructs were observed to lead to endothelial network formation after 14 days. However, to the best of our knowledge, dynamic modulation of stiffness in dECM‐based hydrogels for regulating vascularization has not yet been reported. Indeed, it is challenging to modulate the mechanical properties of pristine dECM hydrogels over a wide range (>200 Pa), even at high dECM concentrations, let alone to dynamically tune the dECM matrix stiffness during cell culture. In contrast, the stiffness of our self‐clickable dSIS‐NB hydrogels can be dynamically tuned not only by adjusting the dSIS‐NB concentration but also by varying light intensity, wavelength, and photoinitiator content, with minimal changes in viscoelasticity (Figures 2 and 3d; Figure S3). While dynamically stiffened self‐clickable dSIS‐NB hydrogels reduced HUVEC network development, additional crosslinking may alter the accessibility of biological ligands (e.g., RGD motif) on fibrillin, such that they are ‘masked’ by the rearranged disulfide bonds and become less accessible to cell surface receptors. However, LAP and light‐induced disulfide exchange may also rearrange the fibrillin protein structure, thereby exposing the otherwise hidden motifs to engage with cell surface receptors. Future studies are needed to elucidate the accessibility of the biological ligands to the encapsulated cells. Nonetheless, these hydrogels enhanced HUVEC spreading and assembly, forming an interconnected network within the 3D fibrous matrices [14, 45]. Furthermore, immobilization of the angiogenic peptide led to only a moderate decrease in gel stiffness, but greatly enhanced network formation even under VEGF deprivation. Hence, self‐clicked dSIS‐NB hydrogels could serve as an in vitro model for studying disease progression, in which altered tissue stiffness affects vascularization. Additionally, other signaling peptide ligands can be immobilized using the same method, permitting the study of ECM ligand‐induced cell fate processes.

### 2.7 Injectable Self‐Clickable dSIS‐NB Hydrogels to Promote Myogenic Differentiation In Vitro

**FIGURE 6:**
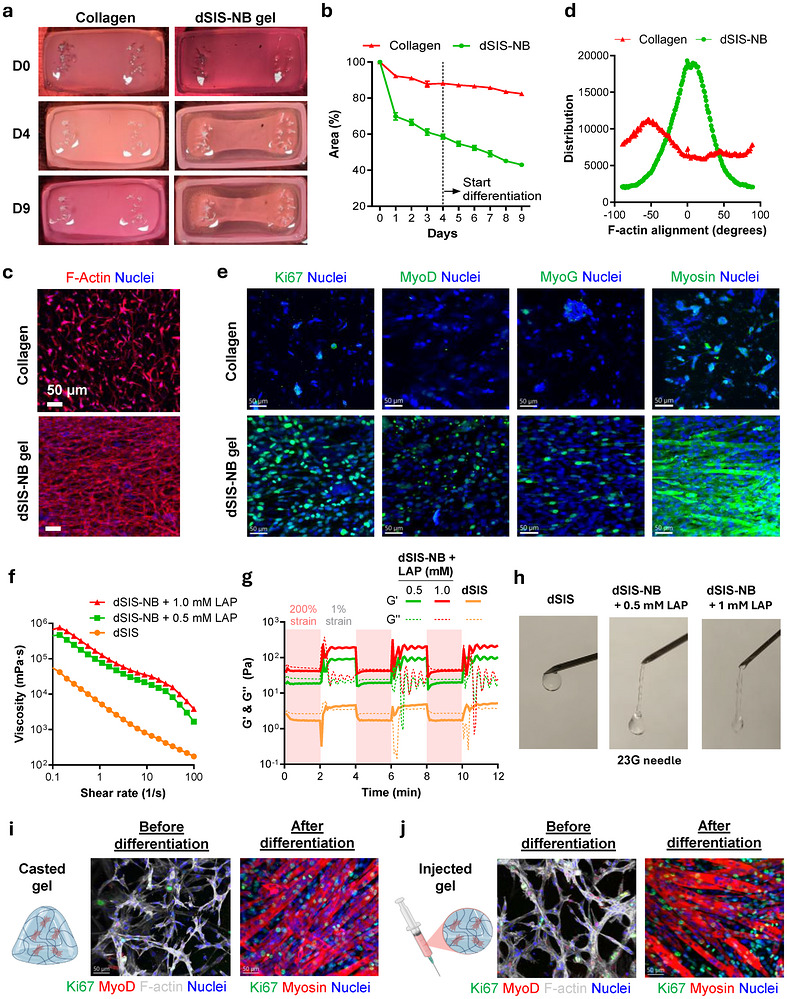
Myogenic potential and injectability of self‐clicked dSIS‐NB hydrogels. (a) Pictures of C2C12‐laden collagen and dSIS‐NB hydrogels. (b) The shrinkage of the hydrogels over time (n = 3, Mean ± SEM). (c) Confocal images of F‐actin staining. (d) Quantification of cell alignment in the hydrogels using F‐actin staining images. (e) Representative immunostaining images of encapsulated cells. (f, g) Viscosity (f) and shear‐thinning measurement (g) using dSIS or self‐clicked dSIS‐NB hydrogels crosslinked by 0.5 or 1 mm LAP (n = 3, Mean ± SEM). Shear‐thinning experiments were conducted with alternate high (200%) and low (1%) strain. (h) Injectability of dSIS and self‐clicked dSIS‐NB hydrogels. (I, j) Immunostaining of C2C12 cells within casted gel (i) or injected self‐clickable dSIS‐NB hydrogels (j) after 4 days of culture (i.e., before differentiation) and after 5 days of differentiation.

We sought to employ self‐clickable dSIS‐NB hydrogels as an injectable cell carrier to treat VML. Thus, we first evaluated the myogenic potential of dSIS‐NB hydrogels. Compared with collagen I gels (G’ ∼85 Pa), C2C12 cells grew in dSIS‐NB hydrogels (G’ ∼ 1500 Pa) induced significant matrix shrinkage (Figure 6a,b) and formed more aligned cellular structures following myogenic differentiation (Figure 6c,d). dSIS‐NB hydrogels also supported much higher levels of Ki67, MyoD, MyoG, and Myosin (Figure 6e), confirming their utility in supporting muscle cell proliferation and myogenic differentiation. Next, we assessed the injectability, viscoelasticity, and shear‐thinning property of self‐clicked dSIS‐NB hydrogels. Owing to the fibrous structure even after photocrosslinking (Figure 1g), the self‐clicked dSIS‐NB hydrogels (crosslinked with 0.5–1 mm LAP) exhibited high viscosity (Figure 6f) and shear‐thinning property (Figure 6g). When injected through a 23‐G needle, unmodified dSIS solution formed discrete droplets without trailing filaments, whereas the self‐clicked dSIS‐NB hydrogels produced smaller droplets that elongated into a long string (Figure 6h), indicating that the pre‐crosslinked gels were still injectable.

To advance the use of the self‐clickable dSIS‐NB hydrogels in muscle regeneration, we evaluated the proliferation and differentiation of C2C12 cells encapsulated in self‐clicked dSIS‐NB hydrogels before and after needle extrusion. The cell‐laden hydrogels were then stained for myogenic markers after 4 days of culture and after 5 days of differentiation (i.e., day 9). The cells spread extensively and formed interconnected networks after 4 days of culture. After 5 more days of myogenic differentiation, the C2C12 cells formed multinucleated myotubes with no distinguishable difference between the two groups (Figure 6i,j; Figure S9a,b). The presence of fibrillin in bovine dSIS‐NB (Figure 1j) may contribute to the enhanced myogenesis via activating ITGA5–ITGB1 signaling, which supports several early steps of myogenesis in C2C12 cells [46]. This pathway includes adhesion, cytoskeletal organization, and directed migration processes that help C2C12 align and initiate fusion. Future studies are needed to elucidate the molecular signaling pathways underlying the enhanced proliferation and differentiation of skeletal progenitors within bovine dSIS‐NB hydrogels.

### 2.8 Injectable Self‐Clickable dSIS‐NB Hydrogels to Promote Myogenic Differentiation In Vivo

**FIGURE 7:**
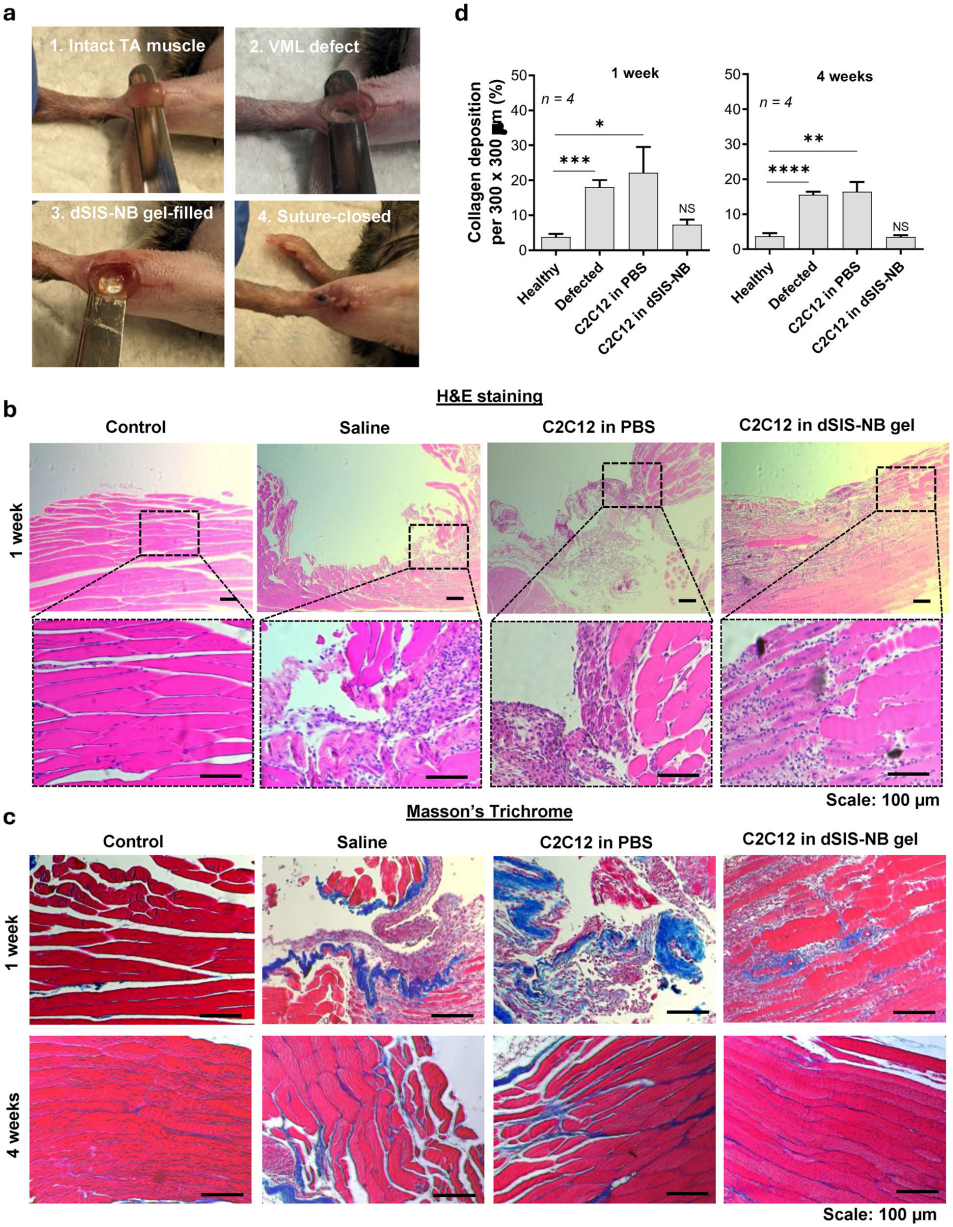
Self‐clickable dSIS‐NB hydrogels promote skeletal muscle regeneration in a VML mouse model. (a) Pictures of mouse VML model with a critical‐size defect, hydrogel implantation (via injection), and suture closure. (b) H&E staining of TA muscle after 1 week of injection. (c) Masson's Trichrome staining of TA muscle after 1 week and 4 weeks of injection. (d) Quantification of collagen area from histology staining images (n = 3, Mean ± SEM. *p<0.05, **p < 0.01, ***p < 0.001, ****p < 0.0001. One‐way ANOVA).

The encouraging in vitro C2C12 encapsulation results prompted us to evaluate the therapeutic effect of self‐clicked dSIS‐NB hydrogels in vivo using a clinically relevant mouse model of VML [5]. A critical‐size defect was first created in the tibialis anterior (TA) muscles of C57/BL6 mice via a 2.5 mm biopsy punch. After the open surgery to create the critical‐size muscle defect C2C12 cell‐laden dSIS‐NB hydrogels were injected at the defect site, followed by suture closure of the wound (Figure 7a). Injection of saline and C2C12 cells in PBS (C2C12‐PBS) served as controls, whereas uninjured muscles served as the positive control. After one week, the uninjured control TA muscle showed normal muscle architecture, whereas the defect sites injected with PBS showed a clear void, and the defects injected with C2C12‐PBS exhibited partial healing (Figure S9c). In contrast, defect sites filled with C2C12‐dSIS‐NB hydrogels demonstrated formation of dense new tissue, suggesting improved integration and early regeneration. H&E staining revealed well‐aligned and intact myofibers in the healthy muscle, whereas the saline‐treated group exhibited severe tissue disruption (Figure 7b; Figure S10). Defect sites implanted with C2C12‐PBS showed partial muscle fiber formation with disorganized morphology, while the C2C12‐dSIS‐NB hydrogels promoted more continuous myofiber alignment as well as myofibers with centrally located nuclei, indicating newly formed myofibers and overall improved tissue regeneration and structural restoration. Masson's Trichrome staining showed densely packed, uniformly aligned muscle fibers with minimal collagen deposition in healthy tissue at week 1 and week 4, whereas saline and C2C12‐PBS groups exhibited disrupted architecture and extensive, blue‐stained fibrotic regions (Figure 7c). In contrast, C2C12‐dSIS‐NB gels formed well‐organized myofibers with markedly reduced collagen accumulation at both time points (Figure 7c), indicating attenuated fibrosis and enhanced muscle regeneration. Although collagen is the major component in dSIS‐NB (Figure 1i,j), Masson's Trichrome staining indicated that the presence of collagen within the dSIS‐NB injected site one week and 4 weeks post‐injection was negligible (Figure 7c,d). Compared with the control, saline, and C2C12‐PBS groups, the collagen signals detected in the C2C12‐dSIS‐NB samples are likely to reflect newly deposited collagen rather than residual dSIS‐NB collagen, which would have been degraded in vivo, as no gel remnants were observed in the explants. These data reinforce the potential of self‐clickable dSIS‐NB hydrogel to promote muscle regeneration.

dECM has been used to regenerate VML [5]. For example, Choi et al. used unmodified muscle dECM as a bioink for 3D bioprinting of cell‐laden gel to repair injured TA muscles in a VML mouse model [44]. However, the bioink was maintained at 18°C to prevent thermal gelation, and a 7 to 14‐day in vitro culture/differentiation period was needed before implantation. In another approach, Nam et al. crosslinked benzaldehyde‐modified polyacrylic acid with gelatin to create a dynamic, self‐adhesive injectable hydrogel for repairing VML [47]. However, this study did not evaluate the myogenic and vasculogenic potential of the acellular hydrogel, and the muscle regeneration capacity was only moderately improved over the untreated control over a 28‐day period. In contrast, the self‐clickable dSIS‐NB hydrogels developed here were viscoelastic and shear‐thinning, enabling their use as a cell‐laden injectable matrix (Figure 7a‐c) for accelerating muscle regeneration in vivo (Figure 7d‐f). The self‐clickable dSIS‐NB hydrogels exhibited injectability, cytocompatibility, and regenerative potential in the VML model. Their shear‐thinning property (Figure 6g) enables extrusion through fine needles while maintaining structure after deposition (Figure 6f). The enhanced muscle regeneration of C2C12‐laden self‐clicked dSIS‐NB hydrogels could be attributed to longer retention of the delivered cells in the defect site, whereas carrier‐free cells might be cleared out quickly upon implantation. Furthermore, the formation of aligned myofibers and reduced fibrosis indicates that dSIS‐NB provides a microenvironment conducive to regenerative remodeling rather than wound closure via scar formation. The reduced collagen deposition seen in Masson's Trichrome staining further supports attenuation of fibrotic remodeling. The degradation of the bioactive dSIS matrix at the injured site may also contribute to regeneration by releasing protein and peptide fragments that facilitate cell proliferation. Future studies are needed to track the retention and proliferation of the injected cells (using fluorescently or luminescently labeled cells), as well as the recruitment and infiltration of the host cells relevant to regeneration. While photo‐crosslinked dSIS‐NB hydrogels are a versatile platform for modulating matrix properties in vitro, their injectability is limited to softer hydrogels. Future work may focus on adapting the self‐clickable dSIS‐NB for enzymatic thiol‐norbornene click crosslinking to enable injectable delivery of stiffer hydrogel formulations. The current dSIS‐NB hydrogels also have limited tissue adhesiveness. This may be improved by incorporating adhesive motifs into the self‐clickable dSIS‐NB hydrogel network, thus creating self‐clickable and tissue‐adhesive double network hydrogels for a wide range of tissue regeneration applications.

## 3 Conclusion

In this work, we introduced dynamic self‐clickable dSIS‐NB hydrogels that overcome limitations of conventional and other chemically modified dECM hydrogels. Through proteomic profiling, we identified fibrillin‐1 as the disulfide‐rich macromolecular component responsible for enabling thiol‐disulfide exchange–mediated photocrosslinking without the need for additional thiolated crosslinkers. This unique fibrillin–norbornene chemistry allows dSIS‐NB to form cytocompatible, nanofibrous, and highly viscoelastic hydrogels that maintain the biomechanical characteristics of native ECM while offering dynamic tunability of stiffness and ligand immobilization. We further demonstrated that self‐clickable dSIS‐NB supports rapid vascular network formation, robust angiogenic sprouting, and dynamic regulation of endothelial morphogenesis through spatially controlled stiffening, softening, and peptide conjugation. Finally, we showed that the injectable dSIS‐NB hydrogels promoted early muscle regeneration, improved tissue filling, and reduced fibrosis in a VML mouse model. Together, these findings establish self‐clickable dSIS‐NB as a powerful advanced dECM that integrates native bioactivity, dynamic covalent chemistry, and tunable mechanics for regenerative engineering.

## 4 Materials and Methods

### 4.1 Materials

Sodium dodecyl sulfate (SDS, #L4390) was purchased from Sigma–Aldrich. Carbic anhydride (#129‐64‐6) and triethylamine (TEA, #21951‐0500) were obtained from Acros Organics. Ellman's reagent (#22582) was purchased from Life Technologies. Fetal bovine serum (FBS), penicillin‐streptomycin (P/S) were acquired from Sigma–Aldrich. 4‐arm PEGSH (PEG4SH, 10 kDa, #155‐163) was obtained from JenKem Technology USA. TCEP‐HCl (#20490) and Fluoradehyde (#26025) were purchased from Fisher Scientific. Paraformaldehyde (PFA, #158127), lithium phenyl‐2,4,6‐trimethylbenzoylphosphinate (LAP, #900889‐1G), and tartrazine (#T0388) were acquired from Sigma–Aldrich. Bovine Serum Albumin (BSA) (#10842‐770) was obtained from Avantor. QK peptide (KLTWQELYQLKYKGIGC, C‐terminal Cysteine residue added for orthogonal thiol‐norbornene conjugation) was custom synthesized and purified to at least 85% purity by GenScript Biotech. Dulbecco's Phosphate Buffered Saline (DPBS, #21‐031‐CV) was acquired from Corning Life Sciences. All other reagents were obtained from Fisher Scientific unless otherwise noted.

### 4.2 Decellularization of Bovine SIS

Decellularization of bovine SIS and synthesis of dSIS‐NB were performed as reported previously [14]. Briefly, a fresh bovine small intestine was obtained from a local grocery. One kilogram of intestine was washed carefully with tap water, then cut into 10 cm in length, and the mesenteric tissues were removed. The intestinal segments were inverted and scrubbed to remove the mucosal epithelium and lamina propria, then returned to their original shape. Tunica serosa and tunica muscularis externa were then removed. The SIS tissues were rinsed with deionized water five times, then with phosphate‐buffered saline (PBS) twice, followed by stirring in 250 mL 1X PBS containing 1% SDS, 2% Penicillin‐Streptomycin, and gentamycin (100 µg mL^−1^) for 4 days, with buffer refreshed every 12 h. The tissues were then vigorously washed in 1000 mL of autoclaved deionized water for 48 h at room temperature, with water refreshed every 6 h. The decellularized SIS (dSIS) was lyophilized at −50°C and 20 Pa for 72 h, weighed, and then cut into small pieces using sterilized scissors.

### 4.3 Synthesis of dSIS‐NB and Quantification of Norbornene Substitution

Hundred‐fifty mg of cut dSIS were sterilely digested in 75 mL acidic solution (0.01 n HCl, pH 2) containing 7.5 mg pepsin at room temperature for 7 days, with constant magnetic stirring (600‐1000 rpm). Next, 135 mg of carbic anhydride (CA) was added to the digested dSIS solution, followed by multiple additions of TEA (135 µL at the beginning, 60 µL after 1 min, 30 µL after 30 min, and 30 µL after 30 min) with magnetic stirring at 1200 rpm at room temperature. This brought the pH to between 7 and 7.5. The mixture was stirred at 800 rpm for 5 h, followed by dialysis in a membrane with a 10–12 kDa molecular cut‐off against precooled autoclaved deionized water at 4°C for 3 days (autoclaved water was refreshed every 12 h). The dried dSIS‐NB was then dissolved in sterile PBS at 1.5 to 2 wt.% using a vortexer at 4°C, stored at 4°C, and used within 1 week.

Fluoraldehyde assay was used to quantify norbornene substitution of dSIS‐NB, with L‐lysine solutions of known concentrations (0 to 0.5 mm) as standards. dSIS and dSIS‐NB were prepared at 1 wt.% in 1X PBS. 20 µL of L‐lysine standard (duplicates) and sample solutions (triplicates) were added to individual wells of a black 96‐well plate. Next, 200 µL of OPA reagent was added to each well, mixed gently with a multi‐channel pipette, and the plate was read on a plate reader with 380 nm excitation and 460 nm emission. NB substitution was calculated using the following equation:

$$\mathrm{Substitution}\;=\left(1-\frac{\mathrm{Amine}\;\mathrm{in}\;\mathrm{dSIS}-\mathrm{NB}}{\mathrm{Amine}\;\mathrm{in}\;\mathrm{dSIS}\;}\right)\times 100$$

### 4.4 Analysis of Protein Compositions in dSIS and dSIS‐NB via Proteomic Profiling

Proteomic analysis was performed by the Indiana Clinical and Translational Sciences Institute (CTSI) Center for Proteomic Analysis. Briefly, 5 mg of lyophilized dSIS or dSIS‐NB samples were resuspended in 40% acetonitrile to a final concentration of 2 to 4 µg/µL. Next, 10 µL of dSIS/dSIS‐NB solution was mixed with 30 µL of 8 m urea. Disulfide bonds were reduced with 15 mm tris(2‐carboxyethyl)phosphine (TCEP) and alkylated with 50 mm chloroacetic acid (CAA). The samples were digested overnight at 35°C with 1 µg trypsin/LysC – (1 h in ∼6 m urea, then diluted to 2 m urea with Tris and added 2 µL PNGase F). The next day, samples were quenched with formic acid and cleaned up with Waters Sep‐Pak cartridge. The samples were resuspended in 40 µL of 0.1% FA and analyzed by the Eclipse‐Aurora column with FAIMS (CID). Norbornene modification sites were identified using PEAKS 12 proteomics software suite by Bioinformatics Solutions Inc.

### 4.5 Analysis of Thiol Contents in dSIS and dSIS‐NB

dSIS and dSIS‐NB were dissolved in 1X PBS at 0.2 wt.% and mixed with TCEP to obtain 0.1 wt.% protein and 0.5 mm TCEP (400 µL). After 15 min at room temperature, oxidized TCEP was removed from the solution using Zeba spin column (#89890, Thermo Scientific) following the manufacturer's protocol. Next, the thiol content of the samples was evaluated using the Ellman assay, with L‐cysteine (0–2 mm) as the standard. Ellman's reagent (#22582, Thermo Scientific) was prepared according to the manufacturer's protocol and added to each well containing the samples and standards (200 µL Ellman's reagent + 25 µL sample/standard). Solutions were mixed gently with a multi‐channel pipettor, and absorbance at 405 nm was measured using a plate reader to determine the free thiol content in the dSIS/dSIS‐NB samples.

### 4.6 Crosslinking and Characterization of dSIS‐NB Hydrogels

Hydrogel precursors were mixed with desired components and kept on ice prior to gelation. Gelation kinetics were evaluated using in situ photo‐rheometry with a modular compact rheometer (MCR 102, Antor Parr) as described previously [48]. A light source (S2000‐XLA, Omnicure) fitted with 365 nm or 405 nm filter was used to initiate gelation under constant strain (1%) and frequency (1 Hz). Typical hydrogel crosslinking was performed by using sandwiched glass slides (0.8 mm gap) pretreated with a water repellent solution. The loaded precursor solution was exposed to light for 2 min. Light wavelength and intensity were indicated in the figures and captions. The moduli of disk‐shaped hydrogels (8 mm diameter, 0.8 mm thickness) were measured using an MCR 102 rheometer in strain‐sweep normal force (NF) control mode with an 8 mm diameter plate. The temperature, NF, shear strain, and frequency for the measurement were 25°C, 0.25 N, 0.1%–0.5%, and 1 Hz, respectively. We utilized shear modulus (G) as a quantitative measure of gel stiffness. The values of storage modulus (G’) are approximately equal to that of G, as the loss modulus (G″) was significantly lower than that of G’. Stress–relaxation tests were conducted by using the MCR 102 rheometer with a strain‐sweep NF control mode and an 8 mm diameter plate. The temperature, NF, shear strain, and frequency for the measurement were 25°C, 0.25 N, 1%, and 1 Hz, respectively.

### 4.7 Microstructure Observations

The microstructure of the hydrogels was observed by Scanning Electron Microscopy (SEM) from Integrated Nanosystems Development Institute (INDI), Indiana University. Hydrogels were prepared via thermal gelation or by photocrosslinking as described in the figure captions. Gel samples were pre‐washed thrice in deionized water, then soaked for 20 min to remove salt and residual chemicals before freezing at −80°C for 30 min and lyophilizing for 24 h under 20 Pa, −50°C. The dried samples were torn out, attached to carbon tape strips, and metal‐coated using Emitech K575X sputter coater and imaged with JEOL JSM‐7800F.

### 4.8 Dynamic Stiffness Tunning

For dynamic stiffening, soft hydrogels (1 wt.% dSIS‐NB) were crosslinked by 0.5 mm LAP under 365 nm light at 8 mW/cm^2^ for 2 min. The initial stiffness was about 200 Pa. The hydrogel was then immersed in a solution containing the desired LAP concentrations for 10 min and then exposed to the light source for 2 min. For dynamic softening, stiff hydrogels (1 wt.% dSIS‐NB) were crosslinked by 6 mm LAP under 365 nm light at 8 mW/cm^2^ for 2 min. The initial stiffness was about 1400 Pa. The hydrogel was then immersed in a solution containing LAP and glutathione (GSH, 5 mm) for 20 min and then exposed to the light source for 2 min. In situ photo‐stiffening and photo‐softening were conducted with the MCR 102 rheometer.

For dynamic photo‐patterning/stiffening of dSIS‐NB hydrogel, soft disk‐shaped (⌀ 10 mm, 0.8 mm thickness) hydrogels (G’ ∼200 Pa) were initially formed using 1 wt.% dSIS‐NB and 0.5 mm LAP (365 nm light, 8 mW/cm^2^, 2 min). The gels were incubated in a solution containing 6 mm LAP and 0.5 mm tartrazine for 10 min, followed by 405 nm light exposure at 34 mW/cm^2^ light source for 60 s, using a circular pattern created in a digital light processing (DLP) bioprinter (LumenX). Dynamic softening of the gels was conducted by incubating the gels in a solution with 6 mm LAP, 0.5 mm tartrazine, and 5 mm Rho‐PEGSH/GSH. The gels were then exposed to 405 nm light at 34 mW/cm^2^ for 60 s. Micropatterns were designed using a free online tool (TinkerCAD), then exported to standard triangle language (STL) files before importing to LumenX.

### 4.9 Endothelial Cell Culture and Encapsulation

HUVECs and endothelial cell medium kits were purchased from ScienCell Research Laboratories (#1001). Red fluorescent protein (RFP)‐HUVECs were purchased from Angio‐Proteomie (#cAP‐0001RFP). VEGF‐free endothelial media was prepared using EGM‐2 endothelial cell growth medium‐2 BulletKit without adding VEGF supplement (Lonza. #CC‐3162). HUVECs were thawed and cultured for 2–3 days at a density of 5000–7500 cells cm^−2^ until they reached ≈70%–80% confluency. Confluent cells were detached using 0.05% trypsin‐EDTA (#15400054, Life Technologies) for 2 min after rinsing twice with sterile PBS, collected, and centrifuged at 500 × g for 3 min. Cells at passages 4–9 were used for all experiments in this study.

HUVEC encapsulation was performed by mixing cells with the hydrogel precursors at about 5 million cells/mL. Fifty microliters of the cell solution were loaded into a sterile glass chamber containing two glass slides treated with water repellent (0.8 mm gap). The chamber was exposed to 365 nm light source for 2 min, then the gels were scooped into a 35‐mm cell culture dish, supplied, and changed media every 2 days. HUVEC seeding on the inner surface of a hydrogel channel was performed by suspending HUVECs in media at ∼10 million cells/mL. Perfusable channel within dSIS‐NB hydrogel was formed by casting the dSIS‐NB precursor around a 23‐G metal needle. The needle was removed after hydrogel crosslinking. Then the HUVEC suspension was infused into the channels using a 10‐µL pipette tip. The hydrogels were incubated at 37°C, 5% CO_2_ for 10 min before being flipped over for homogeneous cell attachment. HUVEC media was refreshed every 2 days. To assess endothelial marker expression, cell‐laden hydrogels were fixed in 4% paraformaldehyde at room temperature for 1 h, followed by permeabilization with 0.2% Triton X‐100 for 1 h. The samples were then blocked in 3% bovine serum albumin (BSA) overnight. Primary antibodies, which were listed in Table S3 were prepared in the blocking buffer, then added to the samples and incubated overnight, followed by 4‐h secondary antibody incubation at RT. 1X DAPI was used to stain nuclei for 1 h. BC34 (Oxford Instruments) confocal microscopes were used in this study.

### 4.10 Vascular Compression Modeling

RFP‐HUVECs were encapsulated within a gel precursor containing 2 wt.% dSIS‐NB + 1 mm LAP at 5 million cells/mL. One hundred microliters of the cell solution were loaded into the sterile 0.8‐mm‐gap glass chamber followed by exposing it to 365 nm light (8 mw/cm^2^) for 2 min. The gels were cultured for 2 days to allow partial vascular network formation, followed by incubation in media containing 6 mm LAP for 10 min and selectively stiffened using a circle‐shaped photomask (2‐mm diameter) under 365 nm light (8 mw/cm^2^) for 2 min.

### 4.11 In Vitro Myogenesis

Immortalized mouse myoblast cell line C2C12 (#91031101 Sigma–Aldrich) were maintained in high‐glucose DMEM (#SH30243.01, Cytiva) with 10% FBS (#35‐010‐CV, Corning) and 1% Penicillin/streptomycin (P/S) (#15240062, Thermo Scientific). Cells within passages 3 to 7 were used in this study. Cells were encapsulated at about 2 × 10^6^ cells/mL in dSIS‐NB hydrogels or neutralized type I collagen (0.8 wt.%, #5226‐20ML, Advanced Biomatrix) as the control. Cell morphology and proliferationwere monitored for 4 days of growth, followed by 5 days of myoblastic differentiation in myogenic media (high‐glucose DMEM + 2% horse serum + 1X insulin‐transferrin‐selenium‐ethanolamine (ITS ‐X) (100X) + 1% P/S). Myogenesis was assessed by immunofluorescence staining as described above, with myogenic markers shown in Table S3.

### 4.12 In Vivo Mouse Model of Volumetric Muscle Loss and Implantation of dSIS‐NB Hydrogels

All procedures involving animals were performed in compliance with the National Institutes of Health (NIH) Guide for the Care and Use of Laboratory Animals and were approved by the Purdue Institutional Animal Care and Use Committee (PACUC protocol #323002368). A total of nineteen, 16–20‐week‐old, male C57BL/6 mice (Inotiv) were used in this study. Mice were anesthetized with 2% isoflurane and administered analgesics (Rimadyl) at 20 mg/kg body weight prior to the surgery. Hindlimbs were shaved using an electric trimmer and Nair hair‐removal cream for 1 to 2 min. The surgical area was cleaned with an antiseptic solution, and a skin incision was made lateral to the tibia using a scalpel. Blunt dissection was performed to separate skin from fascia, and the Tibialis Anterior (TA) muscle was identified. The fascia surrounding the TA muscle was loosened with a scalpel cut, a sterile flat spatula was placed under the TA, and a sterile 2.5 mm diameter biopsy punch was used to create a full‐thickness VML defect in the belly of the TA muscle. There were 4 conditions set up: (1) A control mouse was a healthy mouse (no surgery); (2) Defect mice were mice with VML; (3) PBS + C2C12 mice were mice with VML and injected with C2C12 suspended in PBS at 1 million cell/mL density; (4) dSIS‐NB + C2C12 mice were mice with VML and injected with the preformed dSIS‐NB hydrogel containing C2C12 at 1 million cell/mL density (1 mm LAP, 2‐min, 8‐mW/cm^2^, 365‐nm light exposure were used to preformed the hydrogels). The materials and cells were immediately injected into the defects through a 23G needle, and the skin was sutured back and secured with surgical glue. Both TAs of each mouse were subjected to VML, and group assignments were randomized. Mice were placed back into their cages for recovery. Seven days post‐injection, mice were euthanized, and TAs were harvested for downstream histological processing. Briefly, TA muscles were fixed in 4% PFA for 24 h on a shaker. The samples were infiltrated with paraffin wax, then sliced into thicknesses of 10 µm. The slices were dipped in xylene to remove wax before rehydrating in ethanol and water for H&E and Masson's Trichrome staining. These processes were performed by the Histology Lab Service Core, Indiana Center for Musculoskeletal Health, Indiana University School of Medicine (IUSM). Masson's trichrome images at 10× magnification were loaded into ImageJ (version 1.52P). The images were converted to RGB‐Stack type (Image → Color → Split Channels) prior to threshold setting of the blue channel to display the collagen staining area. Collagen deposition was expressed as the percentage of collagen‐stained areas relative to the total area of the image (300 µm × 300 µm).

### 4.13 Statistical Analysis

The results are presented as the means ± standard error of the mean (Mean ± SEM). All experimental and control groups contained at least 3 independent samples (n = 3) unless otherwise noted in the Figure captions. Comparisons between the two groups were performed using a two‐tailed Student's *t*‐test and One‐way ANOVA in Prism 10 (GraphPad, USA), and a value of *p* < 0.05 was considered statistically significant. ∗ 0.01 < p < 0.05, ∗∗ 0.001 < p < 0.01, ∗∗∗0.0001< p < 0.001, **** p < 0.0001.

## Funding

This project was supported in part by the US Department of Defense (W81XWH2210864) and National Institutes of Health (R01DK127436).

## Conflicts of Interest

None of the authors have a conflict of interest to disclose.

## Supporting information




**Supporting File 1**: advs75296‐sup‐0001‐SuppMat.docx.

**Supporting File 2**: advs75296‐sup‐0002‐VideoS1.mp4.

## Data Availability

The data that supports the findings of this study are available in the supplementary material of this article.
