# Supplementary material for: Dynamic Self‐Clickable Decellularized Matrix Hydrogels for Regulating Vascularity and Enhancing Muscle Regeneration
Source: Adv Sci (Weinh). 2026 Apr 20;13(38):e75296. doi: 10.1002/advs.75296 (PMC13335078; doi:10.1002/advs.75296)
Supplement: Supplementary file 1 — Supporting File 1: advs75296‐sup‐0001‐SuppMat.docx. [file ADVS-13-e75296-s001.docx]

***Supporting information***

**Dynamic self-clickable decellularized matrix hydrogels for regulating vascularity and enhancing muscle regeneration**

Van Thuy Duong,^1^ Tuba Marjan^1^, Ngoc Ha Luong,^1^ Taimoor H. Qazi^1^, and Chien-Chi Lin^1,^ *

^1^Weldon School of Biomedical Engineering, Purdue University, West Lafayette, IN, 47907, USA

*To whom correspondence should be sent:

Chien-Chi Lin, Ph.D.

Professor

Weldon School of Biomedical Engineering

Purdue University, West Lafayette, IN. USA.

Contact Address:

723 W. Michigan St. SL220K

Indianapolis, IN 46202, USA

Phone: (765) 495-7791

Emails: [lin711@purdue.edu](mailto:lin711@purdue.edu)

ORCID: 0000-0002-4175-8796

**Table S1:** Quantification of norbornene (NB) substitution in bovine dSIS-NB.

| **Batch** | **[NB]**  **(mM/wt%)** | **NB substitution**  **(%)** |
| --- | --- | --- |
| 1 | 0.26 | 10.34 |
| 2 | 0.29 | 12.32 |
| 3 | 0.29 | 9.06 |

**Table S2:** Composition of amino acids in bovine Fibrillin I.

| **Amino Acid** | **Number** | **Percent** |
| --- | --- | --- |
| C | 358 | 12.0 |
| G | 336 | 11.2 |
| E | 204 | 6.8 |
| P | 192 | 6.4 |
| N | 187 | 6.3 |
| D | 179 | 6.0 |
| S | 171 | 5.7 |
| T | 168 | 5.6 |
| I | 149 | 5.0 |
| L | 146 | 4.9 |
| R | 146 | 4.9 |
| A | 121 | 4.0 |
| V | 115 | 3.8 |
| K | 114 | 3.8 |
| Q | 104 | 3.5 |
| Y | 94 | 3.1 |
| F | 92 | 3.1 |
| H | 49 | 1.6 |
| M | 48 | 1.6 |
| W | 17 | 0.6 |

**Table S3:** Primary and secondary antibodies (WB: western blot, IF: Immunofluorescence).

| **Primary antibodies** | **Dilution** | | **Supplier** | | | **Cat. no.** |
| --- | --- | --- | --- | --- | --- | --- |
| Phalloidin (Rhodamine) | 1:200 | | Cytoskeleton | | | PHDR1 |
| Phalloidin (670) | 1:200 | | Cytoskeleton | | | PHDN1A |
| Ki67 (rabbit) | 1:200 | | Cell Signaling Technology | | | 9129S |
| MyoD (mouse) | 1:100 | | BD Biosciences | | | 554130 |
| MyoSin (mouse) | 1:200 | | Thermo Scientific | | | 14-6503-82 |
| VE-cadherin (mouse) | 1:50 | | Santa Cruz | | | sc-52751 |
|  |  |  | | | |  |
| **Secondary antibodies** | **Dilution** | | | **Supplier** | **Cat. no.** | |
| Donkey anti Rabbit IgG (H+L) Secondary Antibody, Alexa Fluor 488, Invitrogen | 1:250 | | | Thermo Scientific | A-21206 | |
| Goat anti-Mouse IgG (H+L) Cross-Adsorbed Secondary Antibody, Texas Red-X | 1:250 | | | Thermo Scientific | T-862 | |

**
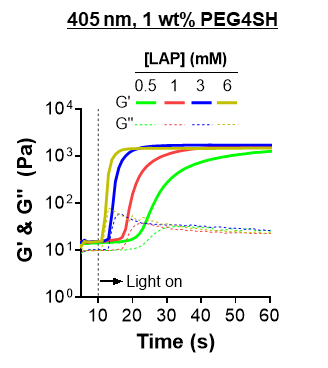
**

**Figure S1:** *In situ* photo-gelation of 1 wt% dSIS-NB with 1 wt% PEG4SH with varying LAP concentration at 405 nm light (8-mW/cm^2^).


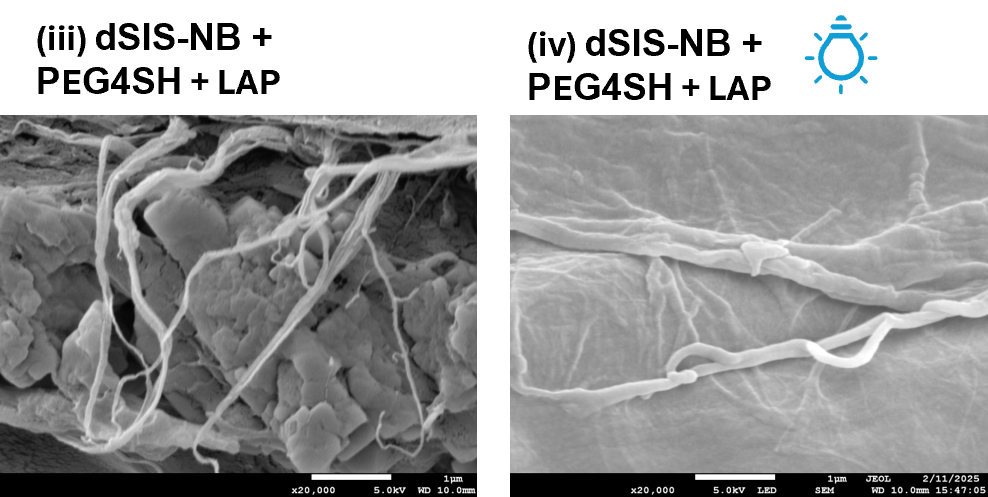


**Figure S2:** SEM magnifications of 1 wt% dSIS-NB photo-crosslinked by 1 wt% PEG4SH and 3 mM LAP (365 nm, 8 mw/cm^2^, 2 min light exposure).


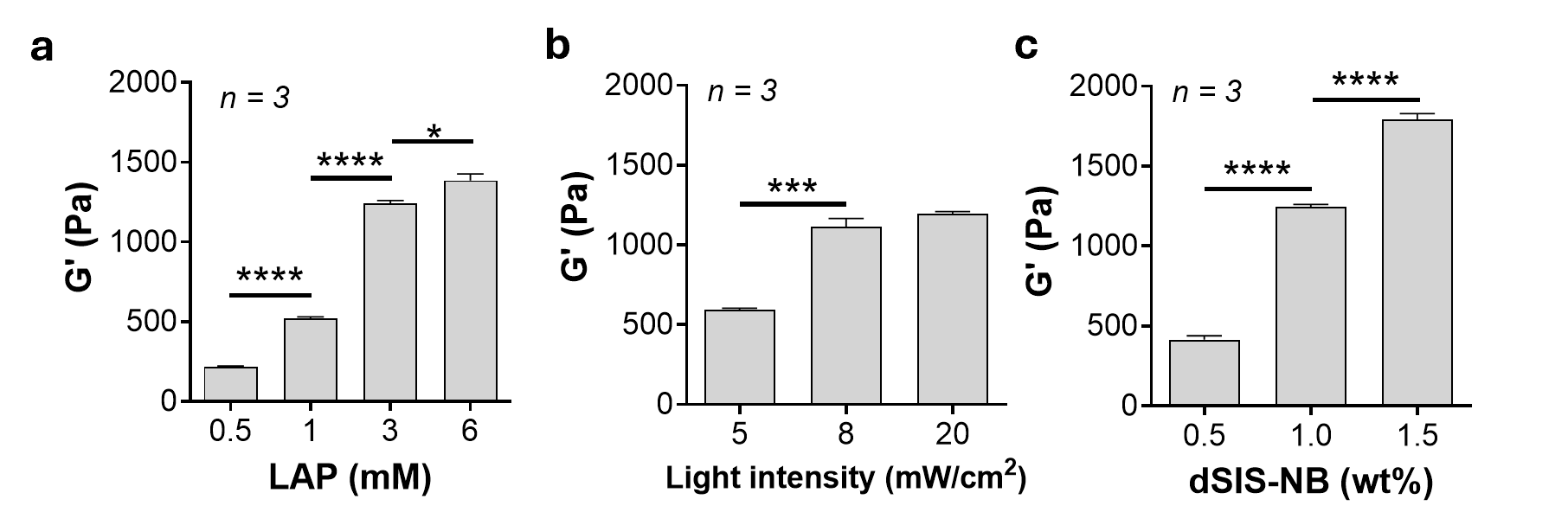


**Figure S3:** Tunability in mechanical stiffness of self-clickable dSIS-NB hydrogels. (a) Storage moduli of 1 wt% self-clickable dSIS-NB hydrogels crosslinked using 365-nm, 8-mW/cm^2^, 2-min light exposure with different LAP concentrations. (b) Storage moduli of 1 wt% self-clickable dSIS-NB hydrogels crosslinked using 3 mM LAP with 365-nm, 2-min light exposure at different light intensities. (c) Storage moduli of self-clickable dSIS-NB hydrogels at different concentrations crosslinked using 3 mM LAP with 365-nm, 8-mW/cm^2^, 2-min light exposure.


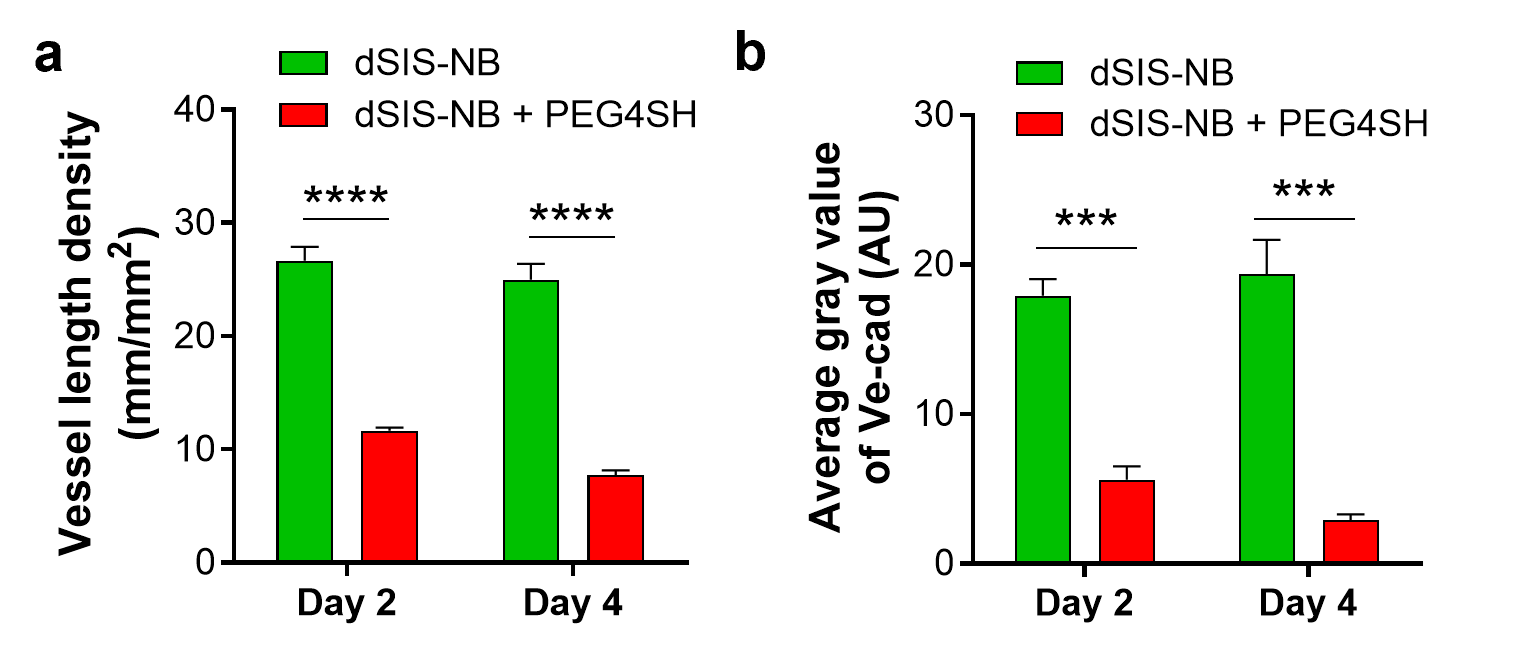


**Figure S4:** Quantification of vascular networks within self-clickable dSIS-NB and dSIS-NB-PEG4SH hydrogels. (a) Vessel length density. (b) Average gray value of Ve-Cadherin.


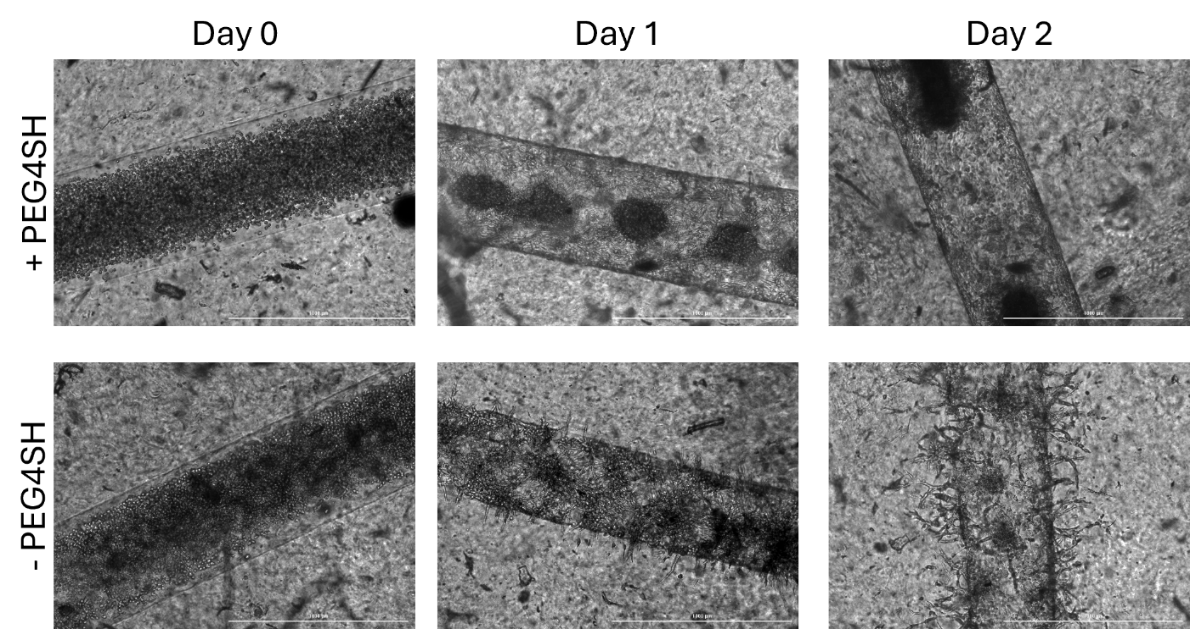


**Figure S5:** HUVEC seeding on the inner surface of perfusable self-clickable dSIS-NB and dSIS-NB-PEG4SH hydrogels.


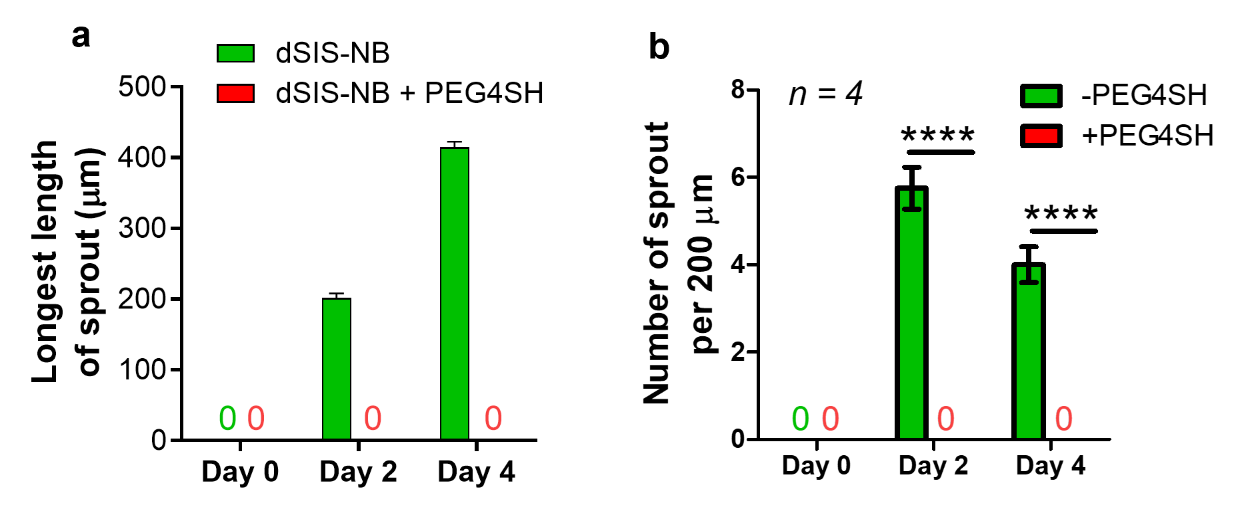


**Figure S6:** Quantification of HUVEC protrusion from channel into bulk self-clickable dSIS-NB and dSIS-NB-PEG4SH hydrogels. (a) Longest length of sprouts. (b) Number of sprout vessel per 200 µm of channel length.


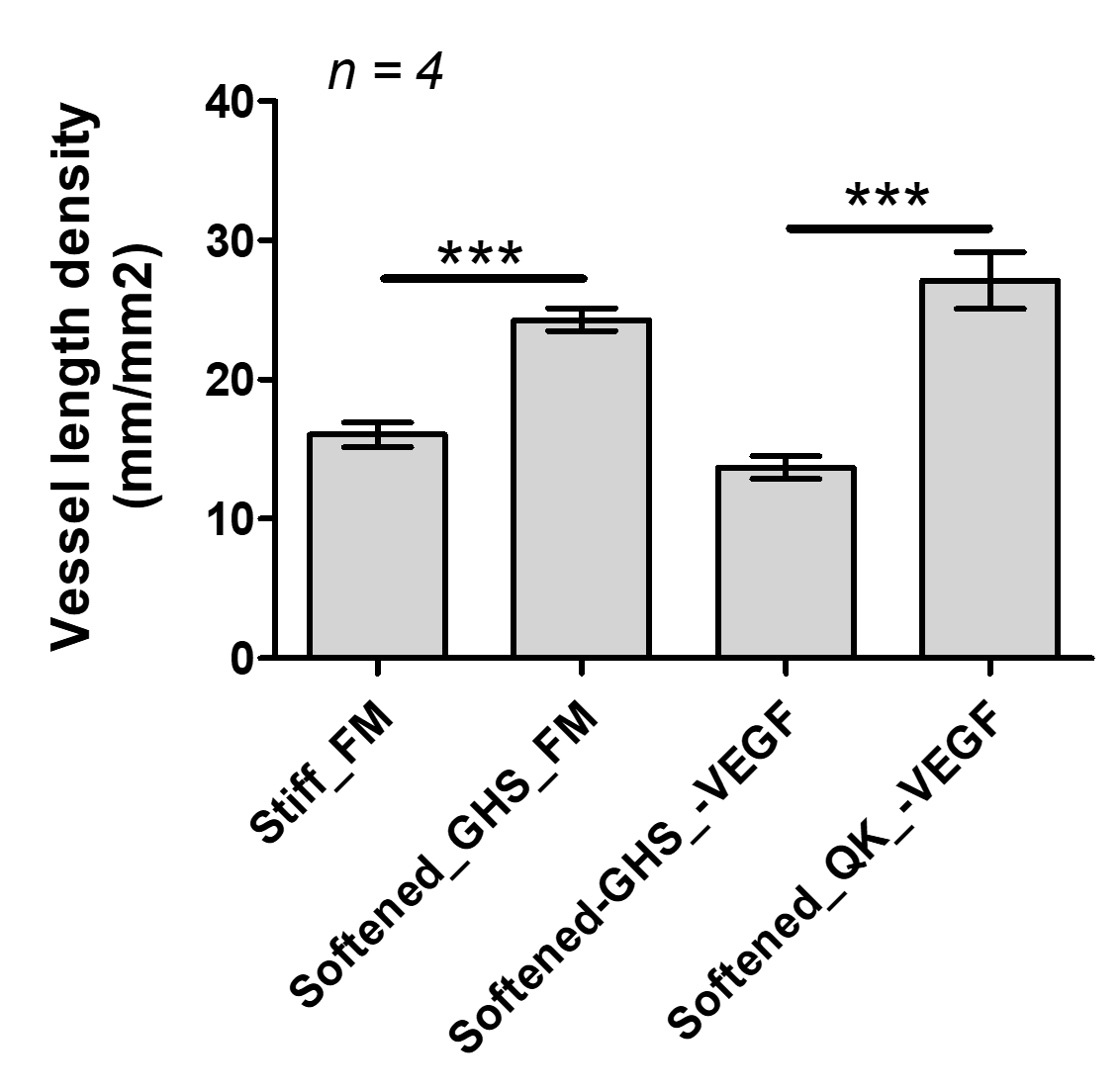


**Figure S7:** Quantification of vessel length density of HUVECs within dynamically softened self-clickable dSIS-NB hydrogels.


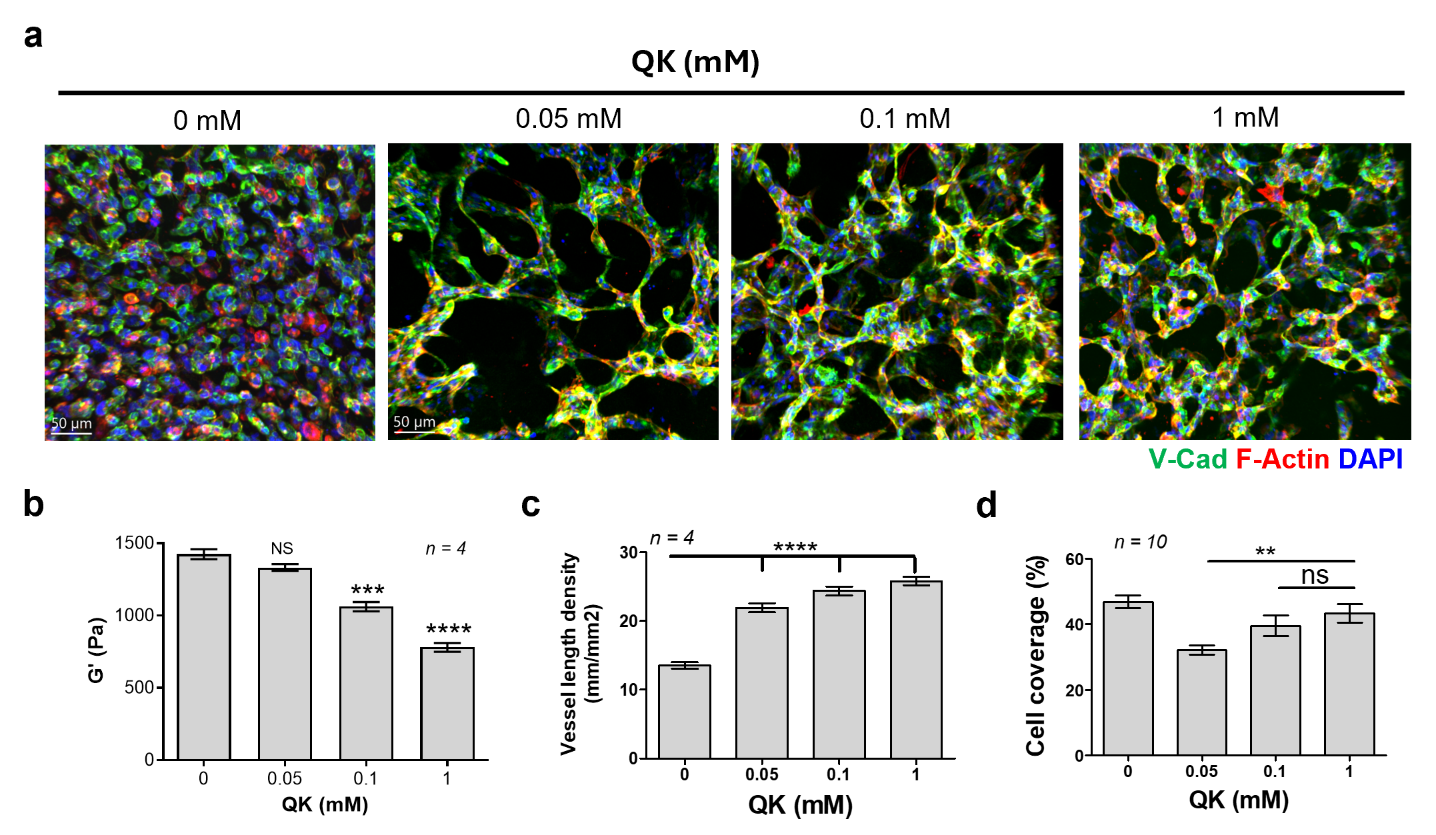


**Figure S8:** Effect of QK peptide concentration on vascular network formation. (a) HUVEC network within self-clickable dSIS-NB hydrogels 2 days post QK peptide tethering at different concentrations. (b) Storage moduli of HUVEC-encapsulated hydrogel right after QK peptide tethering. (c) Vessel length density. (d) HUVEC coverage.


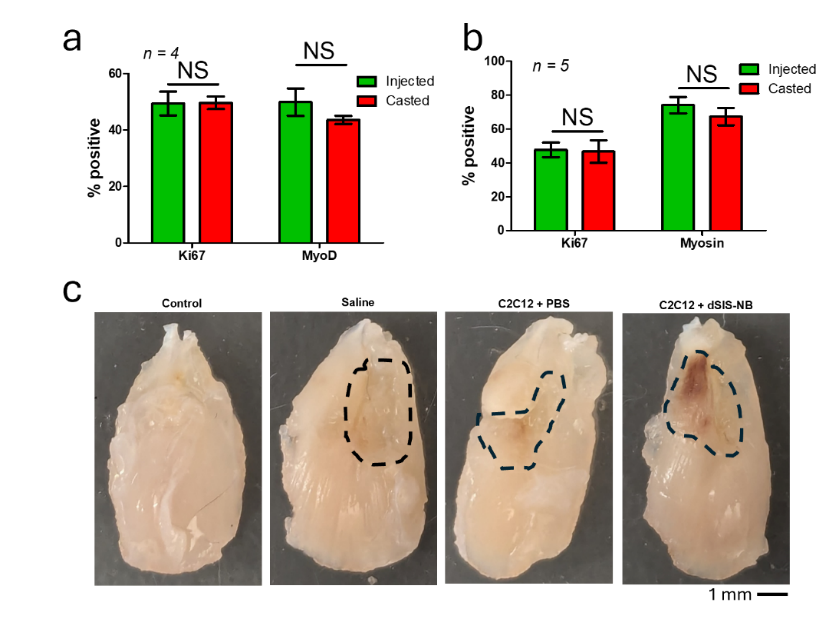


**Figure S9:** Injectability and application of self-clickable dSIS-NB hydrogels in skeletal muscle regeneration. (a) Percentage of C2C12 cells positive with Ki67 and MyoD within 3D casted and injected hydrogels after 4 days of culture in vitro. (b) Percentage of C2C12 cells positive with Ki67 and Myosin within 3D casted and injected hydrogels after 5 days of differentiation in vitro. (v) The overall view of TA muscles from 4 conditions, 7 days after injection.


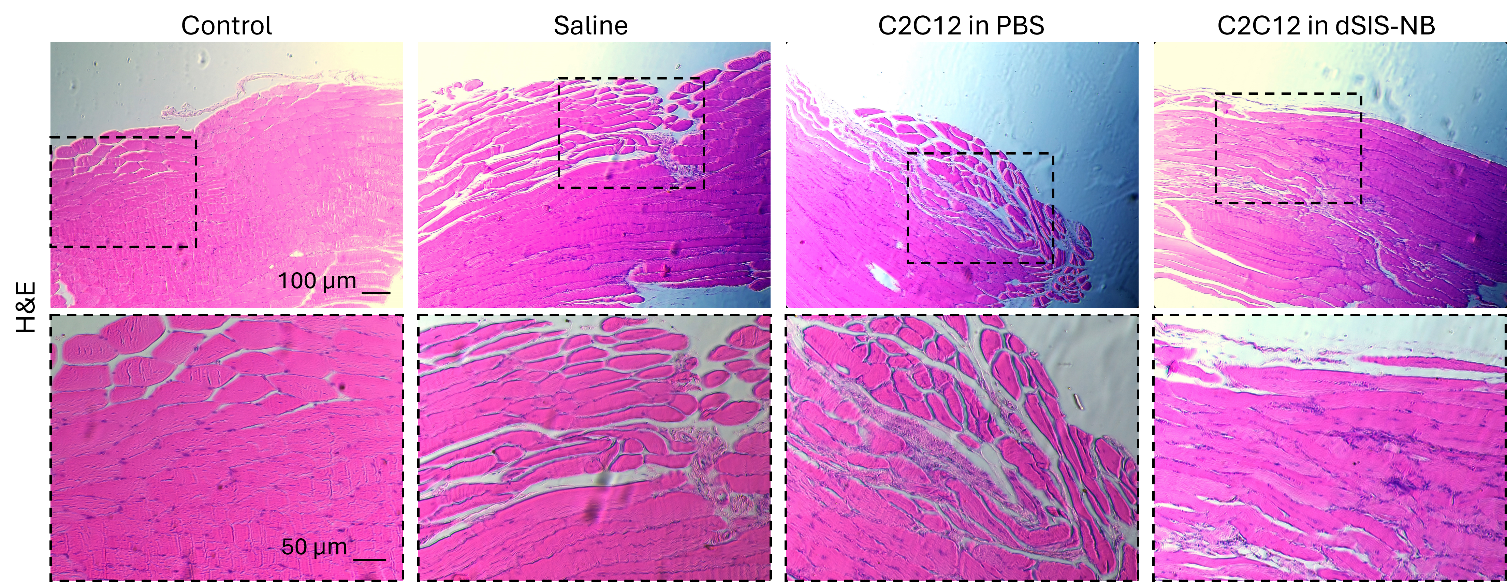


**Figure S10:** H&E staining of TA muscle 4-week post-injection.
